# ﻿Taxonomy, morphology and cytology of *Micranthes virginiensis* (Michaux) Small (Saxifragales, Saxifragaceae), new chromosome counts for *Micranthes* and description of a new *Micranthes* species from the south-eastern USA

**DOI:** 10.3897/phytokeys.267.162132

**Published:** 2025-12-12

**Authors:** Tara K. Hall, Luiz F. L. da Silveira, Max S. Lanning, Katherine G. Mathews

**Affiliations:** 1 Department of Biology, Western Carolina University, Cullowhee, North Carolina, 28723, USA Western Carolina University Cullowhee United States of America; 2 Department of Geosciences and Natural Resources, Western Carolina University, Cullowhee, North Carolina, 28723, USA Western Carolina University Cullowhee United States of America

**Keywords:** Blue Ridge Escarpment, chromosome numbers, hybrid species, *Micranthes virginiensis*, morphology, polyploid, Saxifragaceae, taxonomy

## Abstract

*Micranthes virginiensis* (Saxifragaceae) is an herbaceous, flowering plant species native to eastern North America with a range extending from the Gulf of Mexico into Canada. This broad range, known from previous studies to contain individuals with varying chromosome numbers and morphological variation outside of the current formal description, indicates the need for a re-examination of the taxonomy of this species. Some populations in south-eastern Appalachia display intermediate traits between *M. virginiensis* and the peripatric congener *M. careyana* and have unresolved phylogenetic placement, raising the possibility of hybridisation. This study explored hypotheses of hybridisation and undescribed taxa within *M. virginiensis*, based on morphometric and chromosome data collected from samples across Eastern North America. Floral, fruit and leaf measurements were analysed to investigate morphological variation across the species’ range. Chromosome counts from south-eastern U.S.A. congener populations of *M. careyana*, *M. palmeri* and *M. petiolaris* all showed diploidy (2*n* = 20), representing the first known chromosome counts for these species. Tetraploidy and unique floral morphology in two populations of the Blue Ridge Escarpment of SC, where the distribution of *M. virginiensis* and *M. careyana* abut, indicate an undescribed species, possibly of hybrid origin. Other tetraploid populations in the south-eastern USA showed no morphological differences from diploid *M. virginiensis*, suggesting autopolyploidy. In addition, we document a trend of decreased reproductive investment with increasing elevation within *M. virginiensis.* Overall, the taxonomic boundaries across the broad range of *M. virginiensis* proved intact, aside from the escarpment tetraploid species. Here, we describe the new escarpment species, *Micranthes scopularum* Hall, Lanning & Mathews and provide a complete list of synonyms of *M. virginiensis*, in which we designate two lectotypes and one neotype.

## ﻿Introduction

Geographically widespread species often encompass considerable genetic and morphological variation as a result of adaptations to ecological differences across the range, genetic isolation and drift, migration, selection and genetic sorting (Karron 1987; Soltis and Soltis 1991; Wang et al. 2020). For example, a consequence of environmental heterogeneity can be significant variation in size and reproductive output of individual plants (Cheplick 2005). However, in some cases, variation is representative of undescribed species (Judd et al. 2007; Díaz-Tapia et al. 2018; Ji et al. 2020; Nesom 2021).

One example of a geographically widespread, flowering plant species of Eastern North America with broad-scale morphological variation and multiple ploidy levels is *Micranthes virginiensis* (Michx.) Small (Saxifragaceae), often described as polymorphic (Engler 1872; Johnson 1923) and highly variable (Small 1896; Bush 1928; Lord 1960). Much of the morphological variation noted since the original description (as *Saxifraga virginiensis* Michaux, 1803) has been described at the species and subspecific levels, but most later names have subsequently been synonymised with *Micranthes virginiensis* (International Plant Names Index and external links, https://www.ipni.org/search?q=micranthes%20virginiensis, accessed 11 June 2025). Some exceptional morphological variation has only recently been documented, namely unusual populations found in the Blue Ridge Escarpment region in South Carolina, U.S.A. In this study, we examined both morphological and cytological variation across the entire range of *M. virginiensis*, focusing on plants from the escarpment region, as well as geographical areas from which variants have previously been described, to determine if any constitute unique lineages and deserve species recognition.

Past dramatic fluctuations in climate have resulted in many range changes within the genus *Micranthes*. This has led to disproportionate species richness in cold areas of high elevation and latitude, resulting in evolutionary complexities that remain difficult to disentangle, including hybridisation, cryptic species and variation in chromosome number (Stubbs et al. 2020a). For example, the Pacific North-western *Micranthes hitchcockiana* (Elvander) Brouillet and Gornall (*n* = 38) is believed to be of hybrid origin between *M. rufidula* Small (*n* = 19) and *M. oregana* (Howell) Small (*n* = 19) based on chromosome number and apparent morphological intermediacy (Elvander 1984). The most common chromosome numbers in this genus are 2*n* = 20, 38 and 56, with many species exhibiting several different counts, indicating that aneuploidy and polyploidy are rife throughout the clade (Stubbs et al. 2020a). The multiple instances of 2*n* = 20, 38 chromosomes in *Micranthes* may be a result of chromosome fusions followed by tetraploidisation (Stubbs et al. 2020a). As *Micranthes virginiensis* faces similar complexities to other members of this genus, a study to clarify morphological and cytological variation within the geographic range of this taxon and investigate potential cryptic lineages or hybrid populations would improve our understanding of the evolutionary history and taxonomy of this entity.

Consideration of multiple lines of evidence when drawing species boundaries is crucial in a variable species like *M. virginiensis*, as the morphological, cytological and molecular variations noted in previous studies (Lord 1960; Soltis 1983; Lanning and Mathews 2019) indicate a potential for undescribed and/or cryptic species. This is also true for other members of the Saxifragaceae, as several studies in recent years have delimited new taxa in multiple genera through morphological, molecular and cytogenetic means. For example, *Tiarella* L., a genus long considered to represent only one eastern USA species, *T. cordifolia* L. (Weakley 2020), was recently split into five species, based on previously unnoticed morphological differences in stolon presence and leaf and bract shape (Nesom 2021). In a study examining the utility of nuclear ribosomal DNA sequences for delimiting species, Okuyama and Kato (2009) found evidence of at least three cryptic species in *Mitella* L. and suggested that many other angiosperm lineages contain cryptic species that could be discerned with molecular methods. Additionally, the genus *Tolmiea* Torr. & A. Gray held one species until recently, *T. menziesii* (Pursh) Torr. & A. Gray, containing both diploid and autotetraploid populations. Though the two cytological conditions were not easily morphologically distinguishable from one another, Judd et al. (2007) recognised the diploid entity as a unique species, *T. diplomenziesii*, due to the different geographic distributions and apparent reproductive isolation determined through artificial crossing studies. These studies underline the importance of integrative studies, especially in-depth morphological comparisons, when addressing plant species boundaries.

Although numerous subordinate taxa have been described in *M. virginiensis*, none of them corresponds to populations with known differences in chromosome number. However, polyploidy may have taxonomic significance for this species. Soltis (1983) reported counts of 2*n* = 20 for plants from Massachusetts, Missouri, Tennessee, northern and western Virginia and Canadian populations and 2*n* = 38 in plants from North Carolina and eastern Virginia populations, the latter suspected of autopolyploidisation resulting in tetraploidy (2*n* = 40) followed by an aneuploid reduction (Soltis 1983). Additional counts in this species were done by Hill (2*n* = 20, 1989), Löve and Löve (2*n* = 28, 1982), Kovanda (*n* = 14, 2*n* = 28 as separate meiotic and mitotic counts, 1978) and Löve and Ritchie (1966). The populations of *M. virginiensis* that exhibit polyploidy could be reproductively isolated or associated with unique morphological or ecological characteristics, indicating potential undescribed or cryptic species, as in *Tolmiea*. A variable number of supernumerary chromosomes was also reported by Soltis (1983), which could be of taxonomic significance (D. Poindexter, pers. comm.), further evidencing the complexity of this species. Sister species of *M. virginiensis* (Stubbs et al. 2020b) that adjoin its distribution (*Micranthes careyana* (A. Gray) Small, *M. caroliniana* (A. Gray) Small, and *M. palmeri* (Bush) Bush have not had chromosome counts reported and the potential for hybridisation between *M. virginiensis* and its sister taxa is not known, therefore ploidy levels in this group are in need of further investigation and are a focus of this study.

**Figure 1.**
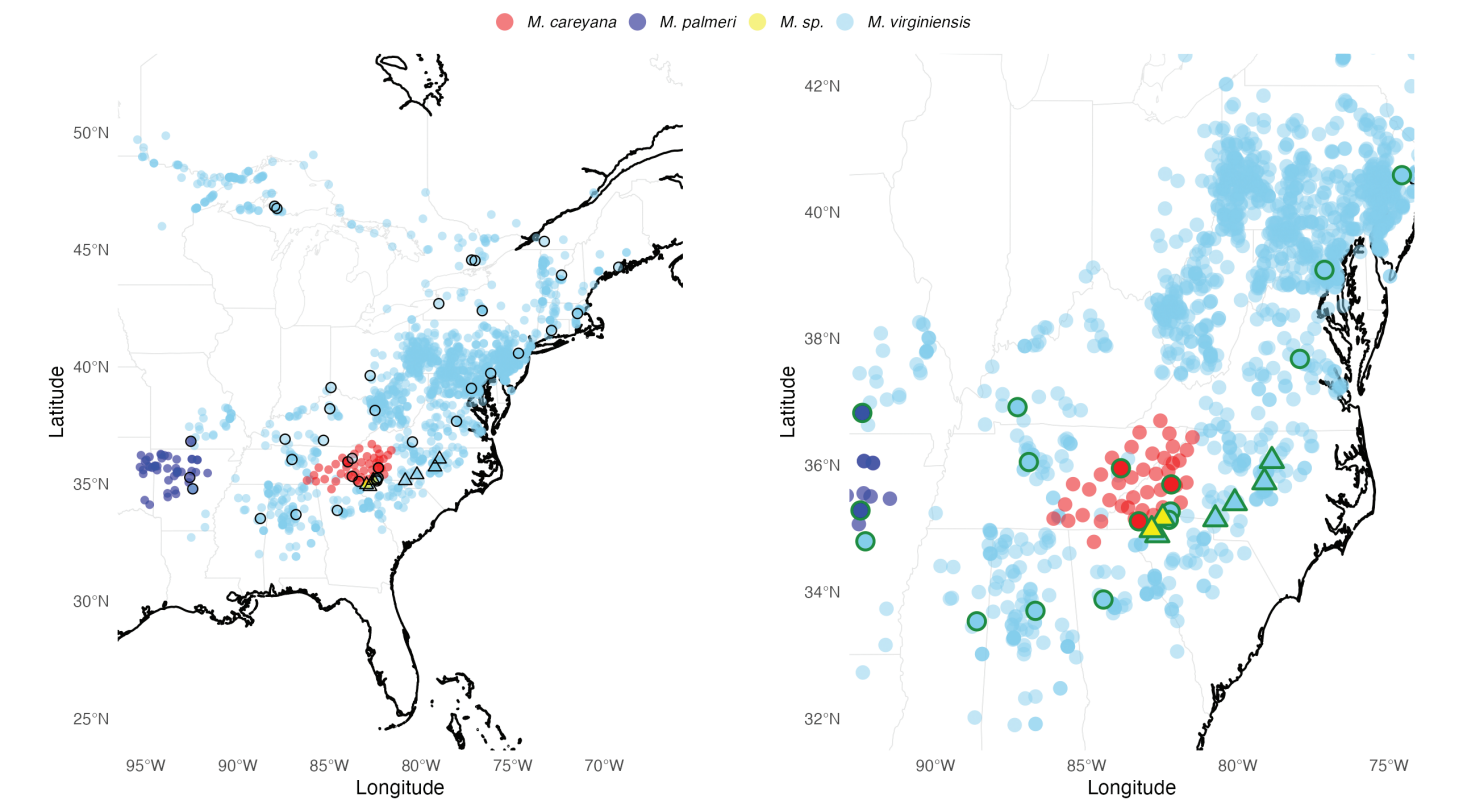
Distributions of *Micranthes virginiensis* and three other south-eastern U.S.A. *Micranthes* species. Total distributions shown in map on the left. Inset map on the right centres on the Blue Ridge escarpment populations. Collections used for chromosome counts in this study with voucher specimens are outlined. Circles indicate diploid plants; triangles indicate tetraploid plants. Collected plants and voucher specimens were also used for morphological observations and measurements.

*Micranthes virginiensis* is found on rock outcrops, moist alluvial and slope forests, streambanks and riverbanks and has a broad distribution from southeast Canada throughout the eastern United States into Louisiana and Arkansas (Weakley (2025); Fig. 1). According to Brouillet and Elvander (2021), it grows at elevations of 0–1500 m, but in the Southern Appalachians, it is restricted to escarpment regions at around 340 m elevation. Lord (1960), who provided the most recent revision of species of *Micranthes* (as *Saxifraga* L.) in the Southern Appalachian Mountains, noted that *M. virginiensis* has long been known to exhibit vegetative variation, particularly concerning leaf shape and margins, distribution of pubescence and scape branching pattern; however, she determined that all individuals she studied were consistent in their presence of a hypanthium (sepals and petals partially fused to and forming a cup-like structure surrounding the ovary) and short stamen length relative to petal length when compared to peripatric *Micranthes* species. However, populations identified as *M. virginiensis*, but inconsistent with these floral characters, have recently been discovered on the Blue Ridge Escarpment of SC (Lanning and Mathews 2019), begging the question of potential undescribed species encompassed under the name. As taxa with cosmopolitan geographic distributions have been frequently shown to hold cryptic diversity (Díaz-Tapia et al. 2018; Wu et al. 2018; Whittall et al. 2020; Nesom 2021), the latitudinal and climatic variation in the distribution of *M. virginiensis* across the eastern United States makes it a likely candidate for harbouring undetected novel taxa.

Hybridisation leading to allopolyploidy could result in hybrid speciation, in which the hybrid offspring can persist and maintain a stabilised hybrid lineage, generally recognised as a species, as this often results in reproductive isolation (Mallet 2007). In addition, many plant studies indicate that polyploid, hybrid species may form recurrently from different progenitor diploid populations and subsequently interbreed to form a polyphyletic species (e.g. Soltis and Soltis (1999); Sonnleitner et al. (2010); Symonds et al. (2010); Sampson and Byrne (2012)). A recent systematic study of *M. caroliniana* and *M. careyana*, two Southern Appalachian endemics, found these species to be phylogenetically closely related and morphologically similar to *M. virginiensis* (Lanning and Mathews 2019) and these relationships were later confirmed by Stubbs et al. (2020b), although they are not known to have the ability to hybridise.

During Lanning’s field collections, two putative *M. virginiensis* populations from Greenville and Pickens Co., SC, in the escarpment region were observed to have some floral characteristics more consistent with *M. careyana*, including long stamens and large fruits and lacking a conspicuous hypanthium, but without the petal spots characteristic of *M. careyana* (vegetatively, *M. virginiensis* and *M. careyana* are indistinguishable). These were included in Lanning and Mathews’ (2019) phylogenetic analyses, based on nrITS and cpDNA gene regions, which resulted in poor resolution amongst populations of these species and *M. caroliniana*, the cause of which was unknown.

The Southern Blue Ridge Province, spanning the Appalachian Mountains from Virginia to Georgia, is known for high rates of endemism and species diversity, including a mixture of species of temperate, tropical and alpine origin and populations at the periphery of many northern and western species’ ranges (Pittillo et al. 1998). This is due to the unique microhabitats created by the geomorphic structure of this region range formed over hundreds of millions of years, as well as the glacial advance and retreat cycle of the past 2 Ma (Pittillo et al. 1998; McMillan et al. 2018). Endemic species, disjunct distributions and morphological oddities are commonplace in the escarpment region (e.g. Gray (1879); Wagner (1965); Billings and Anderson (1966); McMillan et al. (2018)). In fact, a new variety of *Micranthes petiolaris* (Raf.) Small was recently described from Wadakoe Mountain (var. s*healyi* P.D. McMillan & Cushman; Cushman et al. (2020)).

To determine if the escarpment, or any other populations of *M. virginiensis* should be recognised as a separate species, we applied the unified species concept (de Queiroz 2007), which asserts that the only requirement for species status is its existence as a separately evolving metapopulation lineage. Under this concept, multiple properties can be used as evidence of lineage separation, though no one property is recognised as necessary for species status. In this study, quantitative and qualitative morphological differences and ploidy levels were investigated. The use of multiple properties as evidence of novel taxa is valuable in delimiting species within evolutionarily complex groups that have undergone rapid radiation, such as *Micranthes* (Stubbs et al. 2020b).

## ﻿Taxonomic history of *M. virginiensis*

André Michaux described *Saxifraga virginiensis* in his *Flora Boreali-Americana* (1803, p. 269), as follows (translated from Latin): “The whole thing is a little pubescent; leaves oval, obtuse, somewhat petiolate, crenate; scape mostly aphyllous, paniculate, its branches with subsessile alternating flowers; calyx erect”. The range was given as rocky areas of Pennsylvania, Virginia and the mountains of the Carolinas. Michaux cited a drawing in Plukenet’s *Phytographia* (1691, plate 222, fig. 5; https://bibdigital.rjb.csic.es/viewer/13656/?offset=#page=106&viewer=picture&o=bookmark&n=0&q=), but stated that the panicle was not yet fully developed (“*panicula nondum rite explicata*”). Notably, Michaux’s description does not mention the stamens. Stamen length and filament shape are now known to be important characters in delimiting Southern Appalachian *Micranthes* (Lanning and Mathews 2019). The stamens of *M. virginiensis* are short enough to be hidden within the hypanthium and are not readily visible on herbarium specimens, including on the lectotype specimen from Michaux’s herbarium designated by Uttal (1984).

Since Michaux’s original description, many varieties, forms and segregate taxa have been described. Most of these are currently considered synonyms of *M. virginiensis* due to a lack of distinct morphological differences and overlapping distributions. However, two described varieties, S. virginiensis var. californica (Greene) Jeps. (1905) and S. virginiensis var. subintegraGoodman (1950), have been elevated to species status. *Micranthes californica* (Greene) Small (1905) (= Saxifraga virginiensis var. californica (Greene) Jeps. 1901), is a Western North American species that is geographically disjunct from the rest of *M. virginiensis* and morphologically distinguished by differences in petal and sepal shape and orientation (Small 1896). *Micranthes palmeri*Bush (1928) (= *Saxifraga virginiensis* var. s*ubintegra*Goodman (1950)) occurs in Arkansas and Oklahoma, adjacent to the western edge of the range of *M. virginiensis.* It is distinguished from *M. virginiensis* by the entire leaf margins (vs. toothed), lack of glands on the inflorescence pubescence and glabrous pedicels (Steyermark 1959). Molecular phylogenetic analyses have also confirmed these taxa to be distinct lineages (Stubbs et al. 2020b). *Micranthes palmeri* is the sister taxon of *M. virginiensis*, while *M. californica* is less closely related, though still within the core *Micranthes* clade (Stubbs et al. 2020b).

Some authors have recognised segregate taxa from *M. virginiensis*, based on perceived morphological differences associated with particular geographic areas (Sternberg 1810; Hooker 1833; Bush 1928), though these heterotypic species names have all subsequently been synonymised with *M. virginiensis* based on overlap in the characters. Willdenow (1804) described *Saxifraga vernalis* as synonymous with *S. virginiensis* in his original description, automatically rendering the former name as illegitimate in accordance with Article 52 of the International Code of Nomenclature for algae, fungi and plants (ICN, Turland et al. 2025). However, Hooker (1833) re-described *S. vernalis* (Hooker’s name illegitimate as a later homonym of Willdenow’s name) as distinct from *S. virginiensis*, based on differences in the inflorescence — the flower arrangement of *S. vernalis* forms an imperfect corymb or thyrse that contrasts with the sessile, alternate and somewhat unilateral flowers on the branches of the panicle of *S. virginiensis*. Yet, on the plant in Plukenet’s illustration of *S. virginiensis*, the panicle appears to match Hooker’s description and his illustration of *S. vernalis.* In the original description, Willdenow gives the range of *S. vernalis* as Pennsylvania, Virginia, and the Carolina mountains, whereas Hooker ascribes this species to Canada without mention of the range stated by Willdenow and he reports that he received samples of *S. virginiensis* mixed with *S. vernalis* (Hooker 1833). *Saxifraga vernalis* was not considered distinct from *S. virginiensis* by Torrey and Gray (1840) in their Flora of North America, as they noted that they perceived no differences between the two taxa. Hooker (1847) later relegated this taxon to a variety of *S. virginiensis* (as S. virginiensis var. vernalis, also illegitimate).

Sternberg (1810), following Willdenow’s description of *Saxifraga vernalis* as synonymous with *S. virginiensis*, described *S. elongata* as different from *S. vernalis*, based on differences in the inflorescence. *Saxifraga elongata*, occurring in the Carolinas, has an elongated, unbranched scape with a cluster of small branches at the apex that contrasts with the branching scape of *S. vernalis*. Hooker (1833) considered *S. elongata* a variety of *S. vernalis*.

Haworth (1803) first described *Saxifraga pilosa* as a distinct species, based on the pilose nature of the entire plant and the obtusely dentate leaves and he later (1821) indicated *S. vernalis* and *S. virginiensis* as synonyms of his *Dermasea pilosa*. Bush (1928) resurrected the name *Saxifraga pilosa* and recognised it as distinct from *M. virginiensis*, based on morphological and geographic differences. He described *M. virginiensis* as north-eastern, possessing a cyme inflorescence, sharply serrated leaves and multiple scapes, while the southern and mid-western *S. pilosa* was described as racemose, with obtusely dentate leaves and a short, solitary scape (Bush 1928). Steyermark (1959) opposed this split, arguing that, though there is variation in many characters of *M. virginiensis*, there is intergradation of the characters between the two regions and, thus, there is no justification for splitting the species. *Saxifraga vernalis*, *S. elongata* and *S. pilosa* are all currently considered synonymous with *Micranthes virginiensis*.

There are no currently recognised varieties of *M. virginiensis* (FNA vol. 8, 2009), though numerous varieties have been described. Saxifraga virginiensis var. cicinnata Engl. (1872) is described from Pennsylvania and Canada, with the fruiting plants described as loosely paniculate, with elongate secondary branches surpassing the terminal flower and flowers in a cicinnate inflorescence (Engler 1872). Engler cited Hooker’s (1833) narrow interpretation of S. virginiensis as a synonym of his var. cicinnata. Another variety, Saxifraga virginiensis var. cuneata Farw. (1944) was described, based on an apparent discrepancy in the descriptions of *S. virginiensis* in *Gray’s New Manual* (Gray et al. 1908, p. 446) and *North American Flora* (Small 1905, p. 140) — *Gray’s New Manual* describes obovate or oval-spatulate leaves and purplish follicles, whereas *North American Flora* describes ovate, oval or oblong leaves and green follicles. Farwell (1944) observed a population in Keeweenaw Co., Michigan that he felt best aligned with the *Gray’s New Manual* description and designated this S. virginiensis var. cuneata.

Multiple forms of *M. virginiensis* have been described in New England. Within Essex Co., Massachusetts, three forms have been reported to exist in addition to typical *M. virginiensis*, two of which are only known from this County. Saxifraga virginiensis f. chlorantha (Oakes) Fernald (1917) has pale green petals contrasting with the typical white petals, as well as short hairs on the margins and backs of the petals. This form was found in Topsfield, Mass. and only known from a short description (Oakes 1847; Fernald 1917). Saxifraga virginiensis f. pentadecandra (Sterns) Fernald (1917) is apetalous and possesses 15 stamens, as opposed to 10, with five stamens taking the positions of the petals (Sterns 1887; Fernald 1917). It was first described on Manhattan Island in New York, but was later reported in Essex Co., Mass. as well. Saxifraga virginiensis f. glomerulataFernald (1917) is distinguished from typical *M. virginiensis* by a lack of pedicels that cause the flowers to form glomerules and was described, based on three collections by A. S. Pease in Andover, Mass. from 1901–1902 (Fernald 1917). In addition to the Essex Co. forms, Saxifraga virginiensis f. plena Eames differs from typical *M. virginiensis* only in the doubled number of petals (Eames 1931). This form is described from one location in Litchfield County, Connecticut, though Eames noted other reports of double-flowered plants from Massachusetts, Pennsylvania and New York in his original description. Sterns (1887) suggested that these forms were likely teratological phenomena.

Below, we present a list of all accepted synonyms of *M. virginiensis*, listed chronologically, with typification where possible:

*Saxifraga virginiensis* Michx., Fl. Bor.-Amer. 1: 269. Mar 1803. Type: U.S.A. *Haute Caroline et in rupib. Virginiae Pensylvaniae ad Schuykill*, *A. Michaux s.n.* (lectotype, designated by Uttal (1984), p. 35 as “Type”: P! [IDC Michaux, microfiche no. 61, photo 8]). The lectotype specimen was also photographed by Blackwell et al. (2017, 2018) and is available at http://amphoreus.hpcc.uh.edu/botcar/reference_images_2017a/ as file Michaux0563.tif.

*Saxifraga pilosa* Haw., Misc. Nat. 157. Jul-Dec 1803. Type: U.S.A. Kentucky: limestone cliffs on the Ohio 6 miles above Louisville, *C. Mohr s.n*., 6 May 1855. (neotype here designated: US! [US03781685, http://n2t.net/ark:/65665/m3fc3ae0b1-82c8-4615-b5bb-a44db7d3af5a], mixed sheet, plant marked 2). Haworth’s specimens at OXF were mostly thrown away by Fielding (Stafleu and Cowan 1979); no original material could be located at OXF (Serena Marner, OXF, pers. comm.) or in the K digital collections.

*Saxifraga vernalis* Willd., Hort. Berol. 1(4): 43. 1804, nom. illeg. superfl. Type: Hort. Bot. Berol. W., *C.L. Willdenow s.n.* (lectotype here designated: B-W! [B-W08394-010, http://plants.jstor.org/stable/10.5555/al.ap.specimen.b+-w+08394+-01+0]). Willdenow refers to a cultivated plant in the protologue, of which he includes an illustration. The lectotype specimen is incribed “*S. vernalis*” and “Hort. Bot. Berol. W”.

*Saxifraga elongata* Sternb., Revis. Saxifrag.: 9. 1810. Type: U.S.A. *Habitat in Carolina* (holotype: Tab. IV in Revis. Saxifrag.: 9 (1810) [digital image!]). No original specimen that may serve as a lectotype was found among K digital collections where other Sternberg *Saxifraga* specimens reside.

*Dermasea pilosa* Haw., Saxifrag. Enum.: 8. 1821. Type: Based on *Saxifraga pilosa* Haw.

*Dermasea elongata* (Sternb.) Haw., Saxifrag. Enum.: 9. 1821. Type: Based on *Saxifraga elongata* Sternb.

*Saxifraga vernalis* Hook., Fl. Bor. Am. 1: 248. 1829, nom. illeg. (later homonym; Hooker cites *S. vernalis* Willd as a synonym). Type: Hooker cited five syntypes from two localities: “Canada, and to the Mountains,” *Lady Dalhousie. W. Sheppard, Esq. Dr. Richardson. Drummond*; “On the Columbia, and from Fort Vancouver to the Kettle Falls,” *Douglas*. As of this date, none of these specimens has been located at K for lectotypification (Alan Paton, K, pers. comm.).

Saxifraga virginiensis var. vernalis (Willd.) Hook., London J. Bot. 6: 231. 1847. Type: Based on *Saxifraga vernalis* Hook.

Saxifraga virginiensis var. chlorantha Oakes Mag. Hort. Bot. 13: 218. 1847. Type: U.S.A. Massachusetts: Topsfield, 1842 (holotype: unknown, not at GH [Anthony Brach, HUH, pers. comm.], where Oakes’ herbarium was transferred from BSN (Stafleu and Cowan 1979). We decline to select a neotype at this time pending a search for duplicates at other herbaria.

Saxifraga virginiensis var. cicinnata Engl., Monogr. Saxifraga 145. 1872. Type: Engler cited three syntypes from Pennsylvania, Saskatchewan and Montreal, Canada. Engler’s herbarium at B was mostly destroyed (Stafleu and Cowan 1979), although duplicates may exist at other herbaria. We decline to select a neotype at this time pending further investigation to confirm the unavailability of original material.

Saxifraga virginiensis var. pentadecandra Sterns, Bull. Torrey Bot. Club 14: 124. 1887. Type: U.S.A. New York: New York Co. Near High Bridge [about 160^th^ St.], N.Y. Island [Manhattan], Apr 1887, *E.E. Sterns s.n.* (lectotype here designated: NY! [NY03227809, https://sweetgum.nybg.org/science/vh/specimen-details/?irn=4959853]). Two collections are on the sheet, the lectotype is on the right.

*Micranthes virginiensis* (Michx.) Small, Fl. S.E. U.S.: 501. 1903. Type: Based on *Saxifraga virginiensis* Michx.

Saxifraga virginiensis f. elongata (Sternb.) Engl. & Irmsch., Pflanzenr. (Engler) 4, Fam. 117, 1(Heft 67): 41. 1916. Type: Based on *Saxifraga elongata* Sternb.

Saxifraga virginiensis f. cicinnata (Engl.) Engl. & Irmsch., Pflanzenr. (Engler) 4, Fam. 117, 1(Heft 67): 42. 1916. Type: Based on Saxifraga virginiensis var. cicinnata Engl.

Saxifraga virginiensis f. chlorantha (Oakes) Fernald, Rhodora 19: 143. 1917. Type: Based on Saxifraga virginiensis var. chlorantha Oakes.

Saxifraga virginiensis f. glomerulata Fernald, Rhodora 19: 143. 1917. Type: U.S.A. Massachusetts: Prospect Hill, Andover, 24 May 1902, *A.S. Pease 671* (holotype: NEBC! [NEBC00348793, https://plants.jstor.org/stable/10.5555/al.ap.specimen.nebc00348793]).

Saxifraga virginiensis f. pentadecandra (Sterns) Fernald, Rhodora 19: 144. 1917. Type: Based on Saxifraga virginiensis var. pentadecandra Sterns.

*Micranthes pilosa* (Haw.) Bush, Amer. Midl. Naturalist 11(5): 220. 1928. Type: Based on *Saxifraga pilosa* Haw.

Saxifraga virginiensis f. plena Eames, Rhodora 33(392): 169. 1931. Type: U.S.A. Connecticut: Kent, Litchfield County, on a ledge with several similar plants, 3 May 1908, *Hugh Mosher s.n.* (holotype: GH! [GH00575185, https://s3.amazonaws.com/herbaria4/NEVP-Images/2014/2014-03-17-190008/GH00575185.jpg])

Saxifraga virginiensis var. cuneata Farw., Pap. Michigan Acad. Sci. 30, pt. 1: 61. 1944. Type: U.S.A. Michigan: Keweenaw County, Lookout Range, exposed rocks, 13 June 1940, *O.A. Farwell 12254* (holotype: BLH! [BLH0000313, http://plants.jstor.org/stable/10.5555/al.ap.specimen.blh0000313]).

*Spatularia virginiensis* (Michx.) Á.Löve & D.Löve, Taxon 31(2): 345. 1982, as ‘Spathularia’. Type: Based on *Saxifraga virginiensis* Michx.

## ﻿Materials and methods

### ﻿Plant collections

In late winter and early spring of 2022 and 2023, we collected living plant specimens in pre-flowering condition (overwintering rosettes) from 37 populations (at least two individuals per population) of *Micranthes virginiensis*, including the atypical Blue Ridge escarpment populations identified by Lanning and Mathews (2019), hereafter referred to as “Gap Creek” and “Wadakoe Mountain” (Suppl. material 1: table S1). We also collected from populations of other south-eastern U.S.A. *Micranthes* species, including *M. careyana*, *M. palmeri*, *M. micranthidifolia* (Haw.) Small and *M. petiolaris* (Fig. 1; Suppl. material 1: table S1). Plants were potted and grown in the Western Carolina University greenhouse until flowering stalks formed. We removed young flower buds and placed them in vials containing Carnoy’s solution (3:1 95% ethanol:glacial acetic acid) for 24 hours, after which we replaced the solution with 70% ethanol. Vials were placed in a -20 °C freezer for later use in anther squashes for chromosome counts. The potted plants were grown until full anthesis, harvested and pressed as voucher specimens. From late winter to summer of 2022 and 2023, we obtained two living specimens from at least one population of *M. virginiensis* in full anthesis or in fruit from each U.S. State in the range and two Canadian Provinces, Quebec and Ontario (Suppl. material 1: table S1). Southern populations of *M. virginiensis* are known to flower earlier due to the earlier onset of warm weather, so collections began further south and moved northwards throughout the field season to ensure full reproductive characteristics were available for accurate identification. We pressed one voucher specimen from each sampled population and deposited it into the Western Carolina University Herbarium (WCUH).

Newly-collected vouchers and herbarium specimens available through the Southeast Regional Network of Expertise and Collections (SERNEC) Data Portal (sernecportal.org; accessed Feb–May 2023) were georeferenced using the GeoLocate tool embedded in the portal. We then created a distribution map (Fig. 1) for *M. virginiensis* in ArcMap v.10.5 (Esri, Redlands, CA, USA) using all georeferenced specimens, supplemented by additional georeferenced observations from iNaturalist (iNaturalist.org, accessed Feb–May 2023). Due to the volume of SERNEC specimens (1,721 individuals as of May 2023), not every specimen was examined; however, all specimens in peripheral areas of the range and in unexpected locations were thoroughly examined to confirm the identity. Misidentified specimens were digitally annotated and *M. virginiensis* specimens with imprecise locality information were not included in the distribution map or other analyses.

### ﻿Morphological analyses

We dissected live flowers from at least one individual from each sampled population of *M. virginiensis*, *M. careyana* and the putative hybrids from the Blue Ridge escarpment region and imaged them using a Leica M205 C microscope and Leica Application Suite X software (Leica Microsystems, Inc., Deerfield, IL, USA).

Leaf, flower and fruit characters were measured from 69 living and pressed specimens of *M. virginiensis*, the Gap Creek and Wadakoe Mountain escarpment populations and *M. careyana* to compare the morphology of known taxonomically informative characters (see below) and search for additional characters that may be informative to determine if *M. virginiensis* comprises one or more species and if the escarpment populations are intermediate and possibly of hybrid origin (Suppl. material 1: table S1). Characters measured from live flowers included: presence/absence of glandular hairs on pedicel, hypanthium length, petal length, petal width, stamen length, anther colour and pistil length. The largest leaf from each pressed voucher specimen was used to measure: petiole length, blade length, blade width, leaf margin type, blade circularity and blade area. Additional reproductive and vegetative measurements were obtained from 92 fruiting specimen images in SERNEC (Suppl. material 1: table S2) for: fruit length, distance between fruit horns, inflorescence number, scape internode number and length and leaf margin type. Efforts were made to measure the largest flower and fruit from each live plant or specimen image. All measurements of continuous characters were taken in ImageJ (Schneider et al. 2012) from images containing scale bars for accuracy. Leaf shape characters were measured with the LeafJ plugin (Maloof et al. 2013). Finally, non-continuous characters, including branching pattern, inflorescence type and leaf margin type from 155 SERNEC specimen images were examined (Suppl. material 1: table S3).

All multivariate analyses were conducted in R Studio (vers. 4.2.3; R Core Team (2023)). We conducted principal components analyses (PCA) using the *princomp* () function in the stats package (R Core Team 2023) to explore if the specimens form distinct clusters, based on morphological characteristics, identify components responsible for the greatest amount of variation in the dataset and determine if any traits are associated with particular geographic regions. Five PCAs were conducted from the multivariate data matrix: 1) using all continuous floral and vegetative characters for the *M. virginiensis* populations, excluding the putative hybrids from Gap Creek and Wadakoe Mountain, to look for any indication of morphologically distinct clusters, based on a wide range of characters; 2) using only continuous floral characters and plant height (excluding leaf characters) for *M. virginiensis* populations to determine if there are any floral differences that were masked in the first PCA due to similarity in leaf morphology; 3) using only continuous floral characters and plant height (excluding leaf characters) for *M. virginiensis* and the Gap Creek and Wadakoe Mountain populations to determine if individuals from these two escarpment populations would cluster together distinct from *M. virginiensis*; 4) using only continuous floral characters and plant height (excluding leaf characters) for *M. virginiensis*, the Gap Creek and Wadakoe Mountain populations and *M. careyana* to determine if all three groups would cluster separately, based on these characters (previous research has indicated that leaf characters are not taxonomically informative in distinguishing *M. virginiensis* from *M. careyana* (Lanning and Mathews 2019)) and 5) using fruit characters for *M. virginiensis* specimens accessed via SERNEC to determine if fruit size and shape could reveal multiple clusters of *M. virginiensis*. We also explored the relationships between the latent variables (scores) of the PCA and our geographical variables (latitude, longitude and elevation) through correlations and ordinary least squares (OLS) regression models, when the relationships were linear. Putative interactions between predictors were further scrutinised through a permutational multivariate analysis of variance (PERMANOVA), using the *adonis2()* function in the vegan package (Oksanen et al. 2022).

We explored an array of approaches to test our candidate species hypotheses. Two-tailed t-tests were used to determine if the most informative characters identified in the PCAs were significantly different between *M. virginiensis* and the Gap Creek and Wadakoe Mountain populations to investigate the utility of those characters for field identification. We conducted linear discriminant analyses (LDA) with “leave-one-out” cross-validation using the *lda* () function in the MASS package (Venables and Ripley 2002) to determine if the samples of *M. virginiensis*, *M. careyana* and the Gap Creek and Wadakoe Mountain populations can be discriminated, based on the measured characters and identify the characters that best discriminate the samples. We conducted analyses of variance (ANOVA) with the *aov* () function in the stats package (R Core Team 2023) using the LDA scores as latent variables to determine if the groups were significantly different. We used the *adonis2* () function in the *vegan* package (Oksanen et al. 2022) to conduct a permutational multivariate analysis of variance (PERMANOVA) using the morphological data matrix to determine if the three groups are significantly morphologically different. As PERMANOVA cannot distinguish between dispersion and location (Anderson 2017), further analyses were required. A dissimilarity matrix was calculated from the dataset with *vegdist* () and then tested using *betadisper* () to see if dispersion differed significantly amongst groups. We used the ggord package (Beck 2022) to create all figures for the PCAs and LDAs.

### ﻿Chromosome counts

We determined chromosome counts following the procedures outlined in Windham et al. (2020). We removed the young flower buds that had been fixed in Carnoy’s solution and stored in 70% ethanol from the -20 °C freezer and placed them slightly submerged in 70% ethanol in a Petri dish. Under a dissecting microscope, we removed the anthers and broke them open with a needle tip. We separated the tissues from the anthers and used this as material for the chromosome counts. Material was stained on a clean slide with acetocarmine stain and crushed with a dissecting needle with extra acetocarmine added as needed to ensure the sample did not dry out. We added a drop of Hoyer’s solution to increase chromosome visibility and reduce cover slip movement. To finish preparing the slides, we placed a cover slip on and pressed straight down with high pressure for 15 seconds on each corner of the slip as well as the edges. We examined prepared slides with a 65X oil immersion lens using a Leica Stellaris 5 confocal microscope. We created images of cells with countable chromosomes and made chromosome counts for *M. virginiensis*, *M. careyana*, *M. palmeri*, *M. petiolaris* and *M. micranthidifolia* to report new counts, confirm previously reported counts (Soltis 1983; Brouillet and Elvander 2021), detect any geographical patterns of variation in number and compare chromosome numbers in *M. virginiensis* with those from related species and putative hybrid populations. Though attempts were made, we were not able to collect buds of *M. caroliniana* due to its rarity and inaccessibility of sites; thus, its chromosome number remains unknown.

Utilising the root tip squash method, Soltis (1983) noted that, due to the similar size and shape of supernumerary chromosomes and A chromosomes, supernumerary chromosomes of *M. virginiensis* could only be identified during prophase of mitosis, as they appear much darker than A chromosomes. Effort was made in the present study to distinguish between A chromosomes and supernumerary chromosomes, yet it was found that the anther squash method did not allow for supernumerary chromosomes to be distinguished from A chromosomes in any stage of meiosis in these species. As Soltis (1983) reported counts of 2*n* = 20 or 38 chromosomes and 1–6 (and potentially up to eight) supernumerary chromosomes in *M. virginiensis*, we considered any *M. virginiensis* sample with a meiotic count of *n* = 10–18 to be diploid (2*n* = 20 + 0–8 supernumerary) and any meiotic count of *n* = 19–27 to be tetraploid (2*n* = 38 + 0–8 supernumerary).

## ﻿Results

### ﻿Morphological analyses

**Figure 2.**
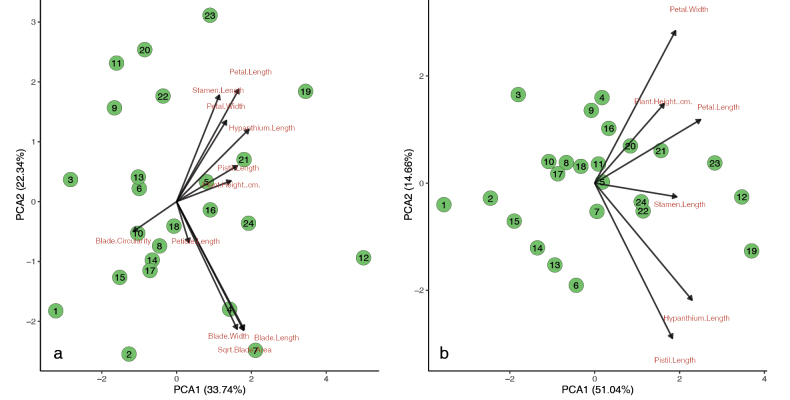
Principal Components Analyses (PCA) of specimens from *Micranthes virginiensis* populations based on morphological data. a. PCA from leaf, flower, and plant height data’; b. PCA from flower and plant height data (excluding leaf characters).

The principal components analysis (PCA), conducted with *M. virginiensis* samples from populations across the range of this species, does not show clusters forming amongst these samples, based on the measured floral and leaf characters (Fig. 2a). This PCA also indicates that the floral character vectors are synergistic and mostly opposed to most leaf character vectors. Both categories of traits are positively correlated with PC1, indicating that these traits increase with size. However, vegetative traits are somewhat negatively correlated based on PC2, indicating a potential trade-off between somatic and reproductive investment in this species.

All traits are negatively correlated with blade circularity along PC1, indicating that, as plants get larger, the leaves become less circular. There is an outlier, data point 12, that is from a West Virginia population that exhibited typical floral and leaf morphology for *M. virginiensis* with the exception of having very large individuals (Fig. 2, Suppl. material 1: table S1). As this sample has a much higher score for PC1, which is associated with size, it is likely that the large size of the individual explains its separation from the other samples.

When exploring the relationships between PCs 1 (~ size) and 2 (~ shape) with the log-transformed geographical variables (latitude, longitude and elevation), we found that somatic (rather than reproductive) investment significantly increases with elevation (Pearson’s *r* = 0.48, p =0.01; OLS’s *ꞵ* = 1.0388, SE = 0.4042, R^2^ = 0.24, *p* = 0.0175; Suppl. material 1: fig. S1A, B) and marginally increases with longitude (Pearson’s *r* = 0.36, p = 0.08; OLS’s *ꞵ* = 8.011, SE = 4.408, R^2^ = 0.1306, *p* = 0.08; Suppl. material 1: fig. S1C, D). A model considering their interaction found only elevation to be a significant predictor of somatic-reproductive allocation (Suppl. material 1: table S6). Interestingly, latitude was never a significant predictor of size or shape (Suppl. material 1: fig. S1E, F).

There are no known leaf characters that can reliably distinguish amongst *M. virginiensis*, *M. careyana* and *M. carolinian*, and preliminary analysis (not shown) indicated that PCAs cluster *M. virginiensis* and *M. careyana* individuals together when leaf characters are included despite their well-documented and consistent differences in floral morphology. As leaf characters appear insufficient to distinguish closely-related Southern Appalachian *Micranthes* species, the remainder of the multivariate morphological analyses excluded leaf characters.

A second PCA was conducted on the same sample set to determine if any clustering would occur, based on floral morphology alone that may have been masked by the inclusion of the leaf characters (Fig. 2b). Once again, no clear clusters form amongst the samples, though the amount of variation explained by PC1 (size) increased from 33.74% (Fig. 2a) to 51.05% (Fig. 2b) and the amount of variation explained by PC2 (shape) decreased from 22.34% (Fig. 2a) to 14.66% (Fig. 2b). This indicates a wide range of sizes of the flowers amongst different individuals, with flower size increasing from left to right, though the flowers generally have similar shape. The specimens did not consistently cluster, based on geographic location with the exception of data points 1, 2 and 3, the individuals with the smallest flower size, which were from populations in the Southern Appalachian escarpment region (not the Gap Creek or Wadakoe Mountain populations, which were excluded from this analysis). Other individuals with small flowers came from Ontario, Michigan and New York (Suppl. material 1: table S1). Notably, the West Virginia individual that was much larger than other individuals in the first PCA falls more within the main group in the second PCA with leaf characters excluded. This shows that the West Virginia individual had much larger leaves than a typical member of this species.

Individuals with relatively large flowers were from Connecticut, Quebec and Arkansas (Suppl. material 1: table S1), indicating that both small-flowered and larger-flowered populations are found throughout the range and are not associated with a particular geographic location. Only two tetraploid individuals (Table 5) were included in this PCA, from Glassy Mountain, Pickens Co., South Carolina (data point 24) and Durham Co., NC (data point 22). These two points clustered together, though their flowers appear to be of average size and shape compared to diploid individuals.

**Figure 3.**
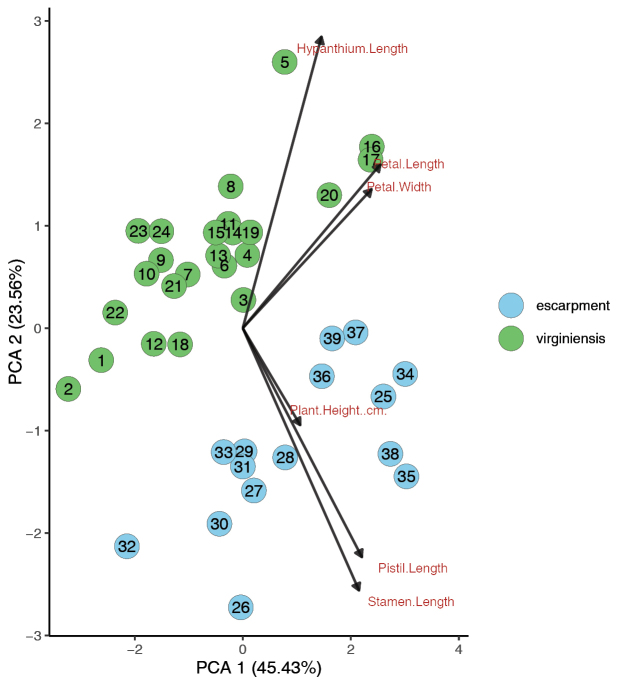
Principal Components Analysis of specimens of *Micranthes virginiensis* and escarpment populations. Escarpment refers to the Gap Creek and Wadakoe Mountain populations. Dataset includes flower characters and plant height.

A third PCA was conducted with floral characters to determine if individuals from the Gap Creek and Wadakoe Mountain populations would be morphologically distinct from the rest of *M. virginiensis* (Fig. 3). Two clusters were observed, one with only samples of typical *M. virginiensis* and one with the Gap Creek and Wadakoe Mountain populations. The Gap Creek and Wadakoe Mountain populations are most associated with the positive vectors for pistil length, stamen length and plant height, though the plant height vector is much smaller than the other vector lengths. This indicates that these plants have longer stamens and pistils and are slightly taller. Though separation between the clusters is visible, there is nearly complete overlap on PC1 and slight overlap on PC2, indicating that, while somewhat different, the two groups are still very morphologically similar.

**Figure 4.**
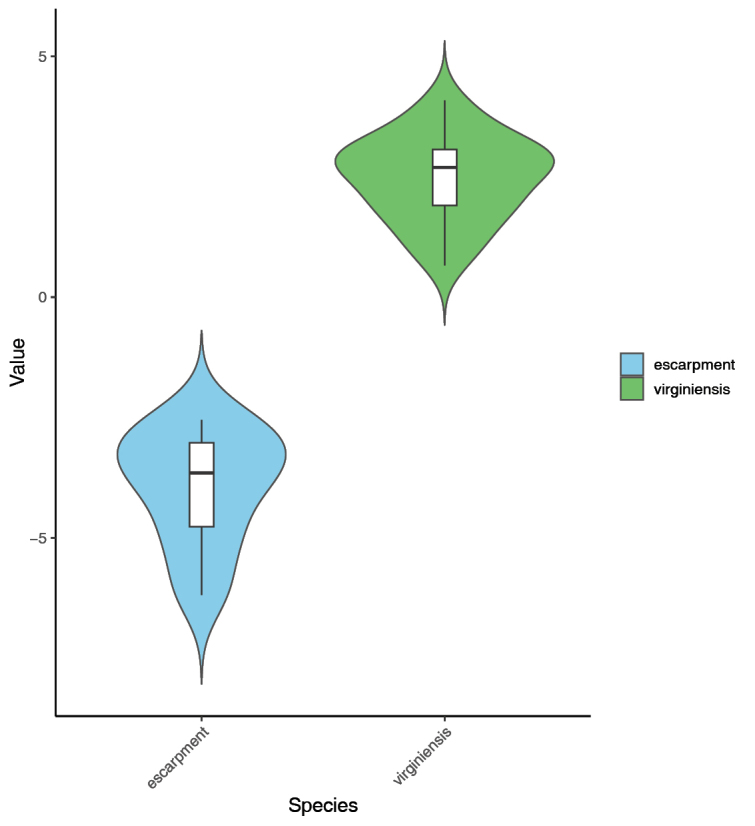
Violin plot depicting scores of an LDA of *Micranthes virginiensis* and the escarpment populations. Escarpment refers to the Gap Creek and Wadakoe Mountain populations. Dataset includes flower characters and plant height.

The LDA (Fig. 4) and subsequent ANOVA conducted using individuals from *M. virginiensis* and the Gap Creek and Wadakoe Mountain populations showed that the measured characters were sufficient to sort the samples into the *a priori* groups (LD1, F = 342.5, *p* < 0.001). All samples were correctly sorted by the model, with the stamen length being the most influential character in discriminating the two groups, followed by hypanthium length (Tables 1, 2). In *M. virginiensis*, hypanthium length ranged from 0.60–1.79 mm (mean = 1.22 mm), compared to the hypanthia of the Gap Creek and Wadakoe Mountain populations, which were smaller on average (0.70–1.36 mm, mean = 1.03 mm). The stamen measurements showed no overlap, with lengths of 0.91–2.04 mm (mean = 1.59 mm) for *M. virginiensis* and 2.24–3.57 mm (mean = 2.90 mm) for Gap Creek and Wadakoe Mountain. Two-tailed t-tests indicated that the two groups have significantly different hypanthium and stamen lengths (*p* = 0.03 and *p* < 0.001, respectively).

**Table 1.** Predicted classifications of *Micranthes virginiensis* and escarpment samples, based on LDA.

	escarpment	*M. virginiensis*
escarpment	15	0
*M. virginiensis*	0	24

**Table 2.** Factor loadings for each character used in LDA of *M. virginiensis* and escarpment samples.

Character	Loading
Hypanthium length	1.09
Stamen length	-2.35
Petal length	0.20
Petal width	-0.03
Pistil length	-0.71
Plant height	-0.29

**Figure 5.**
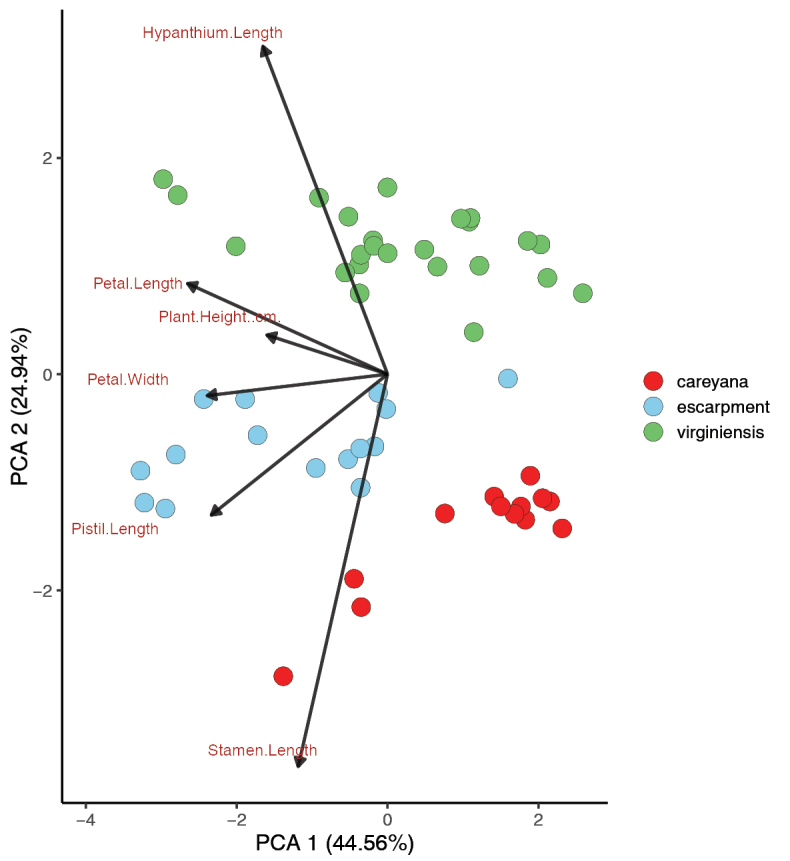
Principal Components Analysis of specimens of *Micranthes virginiensis*, *M. careyana* and the escarpment populations. Escarpment refers to the Gap Creek and Wadakoe Mountain populations. Dataset includes flower characters and plant height.

To visualise clustering between *M. virginiensis*, the Gap Creek and Wadakoe Mountain populations and the other putative parent species, *M. careyana*, a PCA was conducted with individuals from the three groups using floral characters (Fig. 5). Though there is much overlap along PC1, which basically represents overall size, the groups are largely separated by PC2, which mainly summarises the trade-off between the lengths of the hypanthium and stamen. *Micranthes virginiensis* individuals had longer hypanthia and shorter stamens, while the inverse tends to be true for individuals of *M. careyana.* Individuals of the Gap Creek and Wadakoe Mountain populations tend to fall between the *M. virginiensis* and *M. careyana* clusters on PC2, though there is some overlap along this axis between *M. careyana* and the escarpment individuals. It is worth noting that *M. careyana* has distinct yellow/green petal spots, while the other two groups do not (see Fig. 9). As this is a presence/absence trait and not continuous, it is not accounted for in the multivariate analyses conducted in this study.

**Figure 6.**
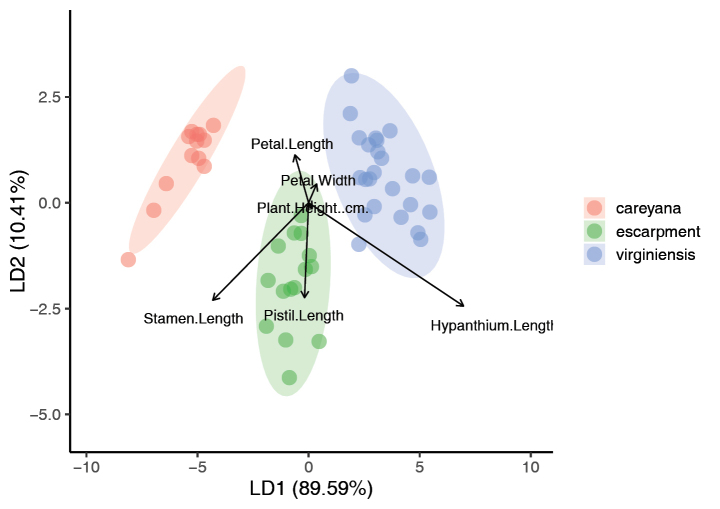
Linear Discriminant Analysis of specimens of *Micranthes virginiensis*, *M. careyana* and the escarpment populations. Escarpment refers to the Gap Creek and Wadakoe Mountain populations. Dataset includes flower characters and plant height. Ellipses represent 95% confidence intervals for the group centroids from the discriminant function.

The LDA (Fig. 6) and subsequent ANOVAs conducted using individuals from *M. virginiensis*, *M. careyana* and the Gap Creek and Wadakoe Mountain populations indicated that the measured characters were sufficient to sort the samples into the *a priori* groups (LD1, F = 335.4, *p* < 0.001; LD2, F = 38.97, *p* < 0.001). The samples were best sorted, based on hypanthium length, which separated *M. careyana* from the other two groups and stamen length, which separated the Gap Creek and Wadakoe Mountain escarpment populations from *M. virginiensis*. These two characters sorted the samples along the first and second axes (LD1 = 89.59%; LD2 = 10.41%), though they were negatively correlated with each other. Pistil length somewhat sorted the escarpment individuals from the other groups on the second axis and the other characters (petal length, petal width and plant height) did not sort the samples at all. The model was able to accurately predict the group for each sample (Table 3). The null hypothesis of PERMANOVA, that the centroid and dispersion are equal amongst groups, was rejected (*p* = 0.001), indicating that the centroid and/or the dispersion are different amongst groups. The dispersion test found no significant differences in the dispersion of values amongst groups (*p* = 0.94), indicating that the groups are significantly different from one another based on location.

**Table 3.** Predicted classifications of *Micranthes virginiensis*, *M. careyana* and escarpment samples based on LDA.

	*M. careyana*	escarpment	*M. virginiensis*
*M. careyana*	13	0	0
escarpment	0	15	0
*M. virginiensis*	0	0	24

An additional PCA conducted with fruit characters and plant height from *M. virginiensis* images from SERNEC (not shown) indicated minimal to no relationship between the size of the fruit and plant height. There are no gaps in the values for fruit length that might indicate multiple distinct groups and fruit size does not appear to be related to geographic area (Suppl. material 1: table S5).

**Figure 7.**
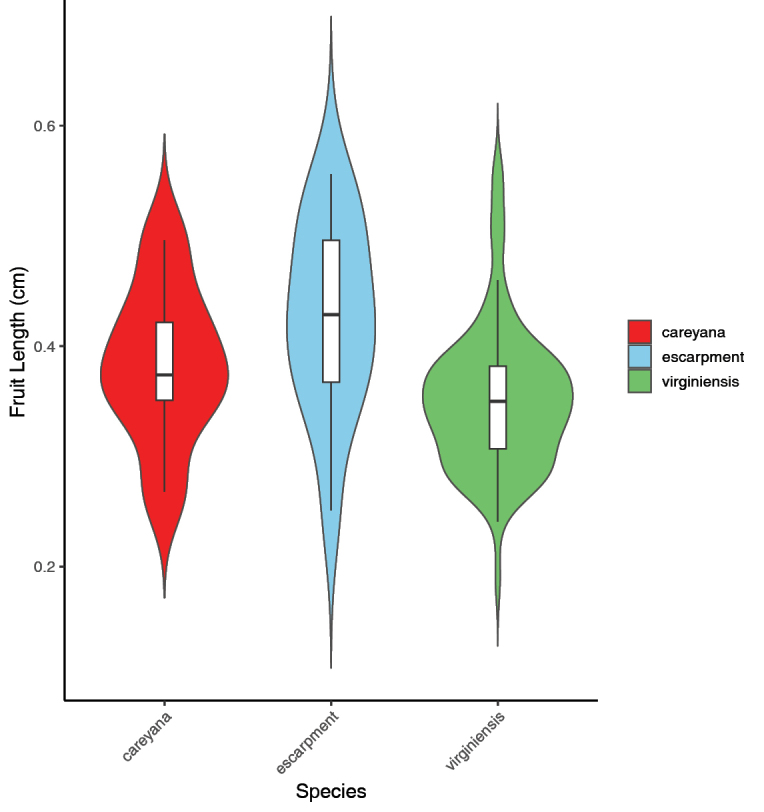
Violin plot comparing fruit lengths of *Micranthes virginiensis*, *M. careyana* and the escarpment populations. Escarpment refers to the Gap Creek and Wadakoe Mountain populations.

Our data indicated that the Gap Creek and Wadakoe Mountain individuals typically have significantly larger fruits than *M. virginiensis* (2.51–5.56 mm, mean = 4.26 mm vs. 1.89–5.59 mm, mean = 3.51 mm; *p* = 0.01); however, there was overlap amongst the samples (Fig. 7). We found *M. careyana* to have fruits of 2.68–4.96 mm (mean = 3.83) and all values fell close to the normal range for *M. careyana* (3–5 mm, mean = 3.61 mm; Lanning and Mathews (2019)). Lanning and Mathews (2019) found that the fruits of *M. careyana* were larger than the fruits of *M. virginiensis*; however, they did not sample from throughout the range of the species. Our data indicated the contrary – *M. virginiensis* fruits are often the same size or even larger than *M. careyana.* The fruit sizes of *M. careyana* and the Gap Creek and Wadakoe Mountain individuals were not significantly different (*p* = 0.294). We summarise the diagnostic morphological characters that may be used to distinguish amongst *M. virginiensis*, *M. careyana* and the escarpment populations in Table 4.

**Table 4.** Diagnostic morphological characters used to distinguish amongst *M. virginiensis*, *M. careyana* and the escarpment populations.

Taxon	Stamen: Petal	Stamen Length (mm)	Hypanthium (mm)	Sepal position at anthesis	Petal Spots	Anther Colour
*M. virginiensis*	< ½ petal length	0.91–2.04	Present (0.60–1.79)	erect	Absent	Yellow
Gap Creek/ Wadakoe	> ½ petal length	2.24–3.57	Present (0.7–1.36)	erect	Absent	Red- orange
*M. careyana*	> ½ petal length	2.56–3.89	Absent	spreading	Present	Red- orange

In all multivariate analyses, individuals from regions containing taxa now synonymised with *M. virginiensis* were found to fall within the primary cluster of *M. virginiensis* samples, confirming the synonymisation, with the caveat that many of these taxa were described, based on discrete characters that were not included in the multivariate analyses, such as branching pattern. After examining discrete characters on herbarium specimens from throughout the range, including all regions from where the synonymised taxa were described, we found no evidence for the recognition of any of these taxa (Suppl. materal 1: table S3).

### ﻿Chromosome counts

**Figure 8.**
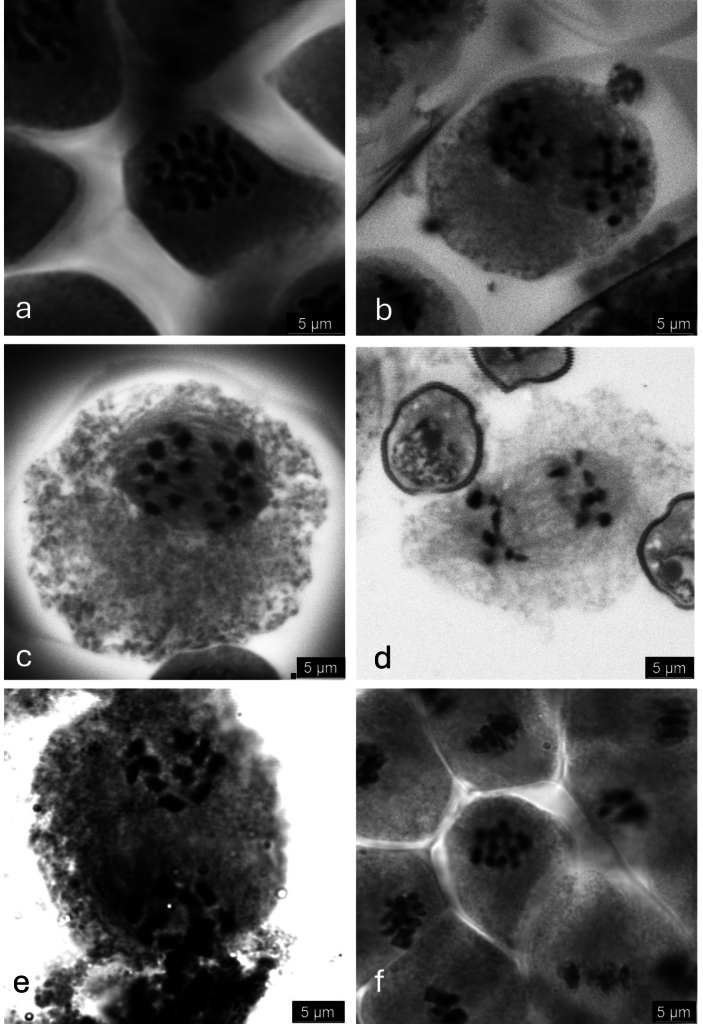
Images of chromosomes in various stages of meiosis from six *Micranthes* populations. a. *Micranthes* sp. *n* = 19 (+1). Greenville Co., SC (Gap Creek). Prophase I; b. *M. virginiensis n* = 19. Chatham Co., NC. Prophase II; c. *M. petiolaris n* = 10. Macon Co., NC. Anaphase I; d. *M. virginiensis n* = 10. Polk Co., NC (Melrose). Anaphase I; e. *M. palmeri n* = 10. Conway Co., AR. Prophase I; f. *M. careyana n* = 10 (+3). Knox Co., TN. Prophase I. Images are from just one plane of focus and, thus, relative sizes of chromosomes may not be accurately reflected in each image.

Chromosome counts were obtained from meiotic pollen mother cells for individuals from 24 populations of *M. virginiensis*, three populations of *M. careyana*, two populations of *M. palmeri*, two populations of *M. petiolaris* and one population of *M. micranthidifolia* (Table 5, Fig. 8). The four populations of *M. virginiensis* sampled from the North Carolina Piedmont region are tetraploid (*n* = 19; Figs 1, 8b). In the Blue Ridge escarpment region, the morphologically distinct Gap Creek and Wadakoe Mountain populations are tetraploid (*n* = 19; Fig. 8a), as is a population consistent with the morphology of typical *M. virginiensis*, the Glassy Mountain, SC population (Fig. 1). In the same region, three morphologically typical *M. virginiensis* populations from this region are diploid (*n* = 10). Sampled individuals from populations from all other geographic regions throughout the range of this species are diploid (Table 5; Figs 1, 8d). In most *Micranthes* populations, supernumerary chromosomes were not observed, though we counted supernumeraries in some populations of *M. virginiensis*, *M. careyana*, *M. petiolaris* and *M. micranthidifolia* (Table 5). With the exception of the Gap Creek and Wadakoe Mountain populations, no morphological differences were observed between diploid and tetraploid *M. virginiensis* in qualitative observations and PCAs.

**Table 5.** Meiotic chromosome counts for populations of *Micranthes* species done in this study.

Species	Voucher	Country	State/Province	County	Chromosome Count *n* (+ supernumeraries)
*Micranthes virginiensis*	Hall 9	USA	AL	Jefferson	10
	Hall 6	USA	AR	Pulaski	10
	Hall 41	USA	CT	New Haven	10
	Hall 10	USA	GA	Fulton	10
	Hall 22	USA	KY	Todd	10
	Hall 38	USA	MD	Montgomery	10
	Hall 39	USA	MI	Marquette	10
	Hall 11	USA	MS	Clay	10
	Hall 1	USA	NC	Polk	10
	Hall 2	USA	NC	Polk	10
	Hall 46	USA	NC	Polk	10
	Hall 5	USA	NC	Durham	19
	Hall 34	USA	NC	Chatham	19
	Hall 50	USA	NC	Mecklenburg	19
	Hall 51	USA	NC	Montgomery	19
	Hall 12	USA	NJ	Somerset	10
	Hall 4	USA	SC	Pickens	19 (+3)
	Hall 19	USA	SC	Spartanburg	10
	Hall 21	USA	TN	Davidson	10 (+1)
	Hall 35	CAN	ONT	Lennox	10
	Hall 7	USA	VA	Powhatan	10
	Hall 36	USA	VA	Floyd	10
*Micranthes* sp.	Hall 3	USA	SC	Greenville (Gap Creek)	19 (+0-4)
	Hall 14	USA	SC	Pickens (Wadakoe Mtn.)	19
*M. careyana*	Hall 31	USA	NC	Macon	10
	Hall 20	USA	TN	Knox	10 (+3)
	Hall 32	USA	NC	McDowell	10
*M. palmeri*	Hall 8	USA	AR	Conway	10
	Hall 16	USA	MO	Douglas	10
*M. petiolaris*	Hall 30	USA	NC	Macon	10 (+0-3)
	Hall 47	USA	NC	Ashe	10
*M. micranthidifolia*	Hall 15	USA	NC	Jackson	11 (+0-1)

We are reporting the first counts for *M. careyana* and *M. petiolaris* as *n* = 10 (+ 0–3 supernumerary) and *M. palmeri* as *n* = 10 (Fig. 8c, e, f; Table 5). For *M. micranthidifolia*, we observed *n* = 11 (+ 0–1 supernumerary) chromosomes (Table 5), agreeing with the previously reported count of 2*n* = 22 (Brouillet and Elvander 2021). Given that many *Micranthes* species are *n* = 10 and that only one population of *M. micranthidifolia* was sampled in this study, it is possible that this species is actually *n* = 10 and that the count reported in this present study and in Brouillet and Elvander (2021) have mistaken a supernumerary chromosome for an A chromosome. However, since there are now multiple accounts of *n* = 11 chromosomes, this should remain the accepted count for *M. micranthidifolia*, though further investigation may be warranted.

### ﻿Taxonomic treatment

#### ﻿ Micranthes scopularum

Taxon classificationPlantaeSaxifragalesSaxifragaceae

Hall, Lanning & Mathews sp. nov.

478AF42E-6B02-5C19-B88C-0B410719BCEC

urn:lsid:ipni.org:names:77373282-1

Figs 9b, c, 10, 11

##### Diagnosis.

*Micranthes scopularum* most closely resembles *M. virginiensis*, but differs in having longer stamens (2.24–3.57 mm long vs. 0.91–2.04 mm long) that are greater than half the length of the petals (vs. less than half the length of the petals), that are exserted from the petals (vs. included within the petals) and red-orange anthers (vs. yellow).

**Figure 9.**
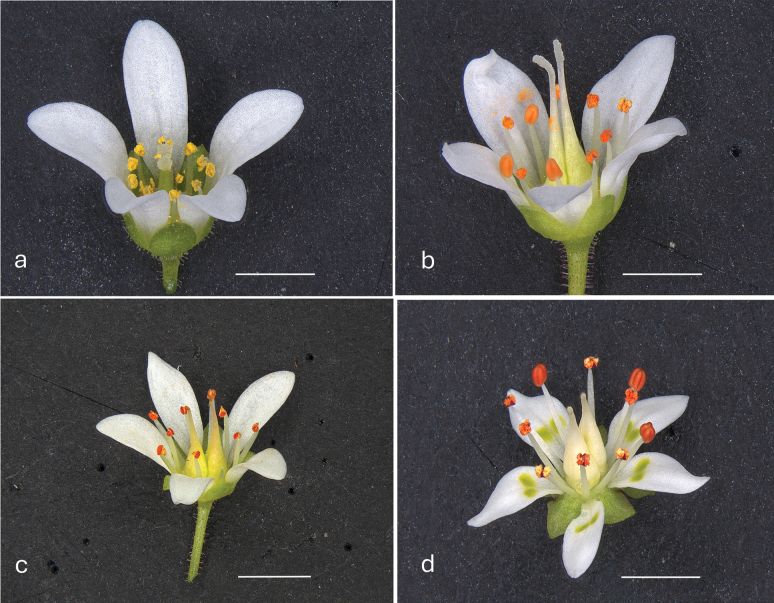
Comparison of flowers of *Micrathes scopularum* and related species. a. Typical *M. virginiensis* flower. Glassy Mountain, SC.; b. *M. scopularum* flower. Gap Creek, Greenville Co., SC.; c. *M. scopularum* flower. Wadakoe Mountain, Pickens Co., SC.; d. Typical *M. careyana* flower. Swain Co., NC. Scale bars: 2.5 mm.

**Figure 10.**
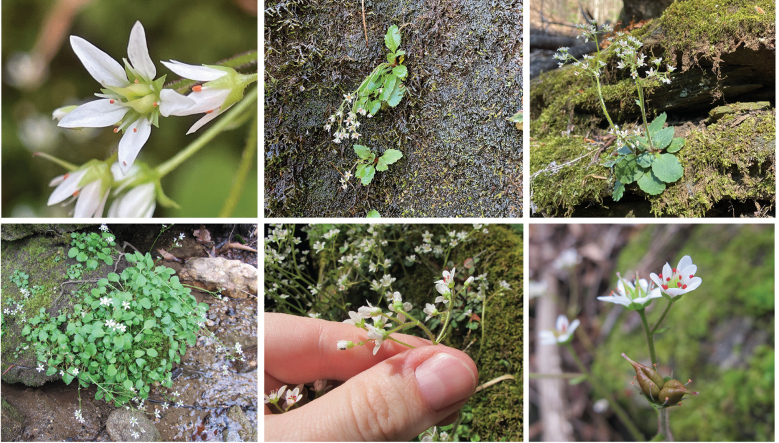
*Micranthes scopularum* plant and habitat photos. From upper left to right: Flowers at anthesis, showing spreading, unspotted petals, exserted pistils and stamens and red-orange anthers; plants growing on bryophyte-covered, seepy, vertical rock face at type locality on Wadakoe Mountain (photos by S. Tessell); plants showing basal rosettes and lax scapes. From lower left to right: cluster of plants growing on moss-covered rock above creek at Gap Creek locality; flowers with hand for scale; part of inflorescence with flowers in side view showing upright sepals and short hypanthium and a developing fruit showing the 2-carpellate, follicle-like capsule (photos by M. Lanning).

##### Type.

**Figure 11.**
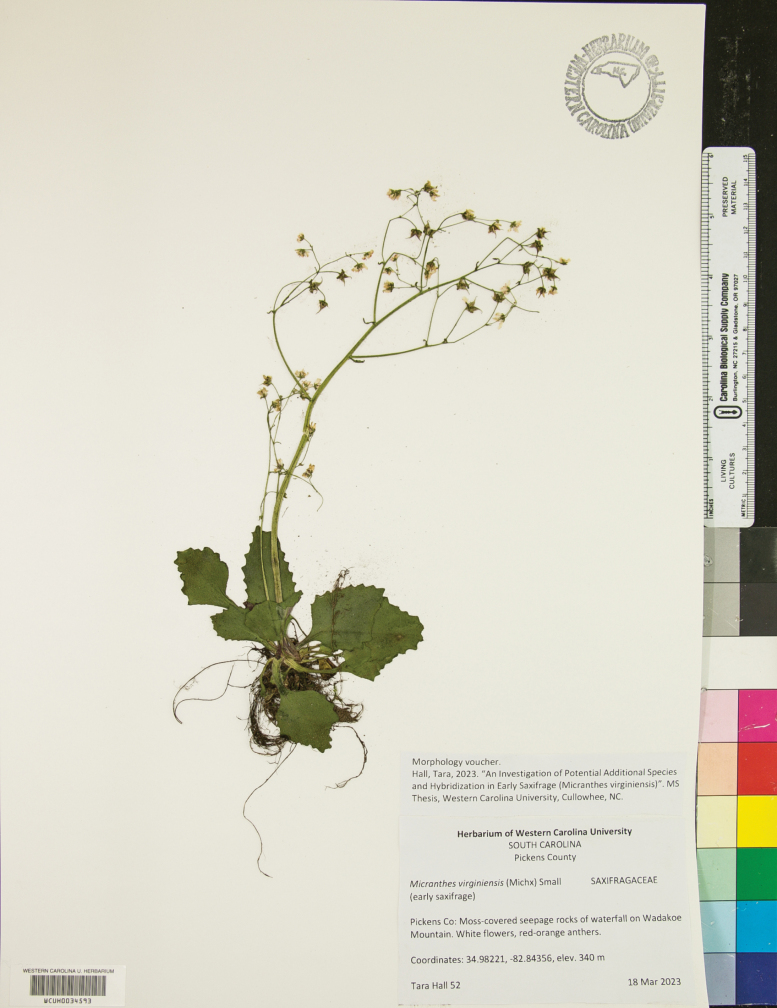
Holotype specimen of *Micranthes scopularum*.

United States of America. South Carolina, Pickens County, moss-covered seepage rocks of waterfall on Wadakoe Mountain, 34.98221°N, 82.84356°W, elev. 340 m, 18 Mar 2023, fl., *Tara Hall 52* (Holotype: WCUH (WCUH0034593; Fig. 11); isotypes: CLEMS, GA, NCU, WCUH).

##### Description.

Acaulescent, short-rhizomatous ***herbs***; ***caudex*** scaly; ***roots*** fibrous.

***Flowering stems*** erect, leafless, 12–26 cm high.

***Leaves*** membranaceous, basally disposed, petiolate; ***petioles*** (0.3–)0.7–2.8 cm; ***blade*** shape variable, ovate, elliptic, orbicular, quadrate or obovate, 0.9–4.2 cm long, 0.7–2.3 cm wide, upper surfaces sparsely hispid or strigose with uniseriate trichomes, lower surfaces pubescent with multiseriate trichomes; ***apices*** acute to obtuse, ***bases*** cuneate to truncate; ***margins*** crenate to serrate, teeth mucronulate.

***Inflorescences*** lax, bracteate thyrses with monochasial subunits, with stipitate-glandular hairs on scape, branches and pedicels; ***bracts*** linear to elliptic, 0.6–2 cm long; ***bracteoles*** linear, 2–6 mm long.

***Flowers*** radially symmetric, pedicellate; ***pedicels*** 3–20 mm long; ***hypanthium*** ca. 1 mm long; ***calices*** green, shortly synsepalous, ***sepals***, 1–2 mm long, broadly ovate, apices obtuse, erect to barely spreading at anthesis; ***corollas*** apopetalous; ***petals*** white, lacking spots, oblong, spreading at anthesis, 3.0–5.0 cm long, 1–2 cm wide, apices obtuse.

***Stamens*** 3–5 mm long; ***filaments*** tapering to the apex; ***anthers*** reddish-orange.

***Gynoecium*** superior, bicarpellate, glabrous, 3.5–5.0 mm long; ***ovary*** basally syncarpellous; ***styles*** and stigmas 2, slightly divergent; ***stigmas*** capitate.

***Capsules*** smooth, green, the 2 valves follicle-like, widely divergent, fused only at base, dehiscing introsely, each valve 3.5–6.0 mm long, 2.0–2.5 mm wide, styles and stigmas persistent; ***seeds*** numerous, brown, ellipsoid, tuberculate, ca. 0.5 mm long.

##### Etymology.

The epithet means of cliffs and rock faces, as found on the steep edge of a plateau, also known as an escarpment, the landform on which the new species is found.

##### Distribution, habitat and ecology.

This species is known from two populations on the southern Blue Ridge Escarpment in northwest South Carolina, ca. 340 m elevation, where it grows on moss-covered rocks within a rich cove forest on Wadakoe Mountain, both on private property and within a State Heritage Preserve and Wildlife Management Area in Pickens County, SC. and on mossy rocks in and around Gap Creek, near Marietta, within the Mountain Bridge Wilderness Area and Jones Gap State Park in Greenville County, SC.

##### Phenology.

Flowers from late February to early April and fruits in mid-April to early May.

##### Preliminary conservation assessment.

At this time, there are only two known populations of *Micranthes scopularum*, including Wadakoe Mountain, in Pickens Co., SC. (the several collection localities on the mountain are assumed to constitute the same population) and the Gap Creek watershed in Greenville Co., SC. Population sizes appear stable over the years that we have observed them, but numbers are unknown. Each population is located, at least in part, on state-owned public land protected and managed by the South Carolina Department of Natural Resources. The Gap Creek population was impacted by fallen trees and floodwaters caused by Hurricane Helene, which devastated many parts of this region on 26–27 September 2024, but the severity of the impact is unknown.

##### Paratypes.

**U.S.A. South Carolina**: **Greenville Co.** • Marietta: Gap Creek, ca. 0.25 mi (0.8 km) down Gap Creek Road off U.S. Route 25. 35.1641220°N, 82.475519°W. 18 Mar 2023, fl., Tara Hall 48, 54, 55, 56, 57, 58, 59 (CLEMS, NCU, WCUH). [same location] 6 May 2023, fr., Tara Hall 49, 74, 75, 76, 77, 78, 79, 80, 81, 82 (CLEMS, NCU, WCUH). **Pickens Co.** • Moss-covered seepage rocks of waterfall on Wadakoe Mountain (private), 34.98221°N, 82.84356°W, elev. 1114 ft (340 m), 19 April 2008, fr., M. Lanning 18 with P. McMillan • Cooler Property, seep on N-facing slope of Wadakoe Mountain, 34.98252°N, 82.8441°W, elev. 332 m. 18 Mar 2023, fl., S.M. Tessel 23031801 (CLEMS) • Jocassee Gorges Wildlife Management Area, west fork of ‘Cooler Creek’ on N slope of Wadakoe Mountain. 34.98203, -82.84414, elev. 348 m. 18 Mar 2023, fl. S.M. Tessel 23021802 (CLEMS).

### ﻿Identification key to *M. scopularum* and related species

The following key has been revised from Lanning and Mathews (2019) to include the new species:

**Table:** 

1	Sepals completely reflexed at anthesis; filaments clavate (use 10×)	***M. caroliniana***
–	Sepals upright to spreading at anthesis; filaments tapering to the apex (use 10×)	**2**
2	Stamens included, 0.9–2.0 mm long, less than half the length of the petals; anthers yellow	***M. virginiensis***
–	Stamens exserted, 2.2–3.9 mm long, greater than half the length of the petals; anthers red-orange	**3**
3	Hypanthium absent; petals and sepals spreading at anthesis, petals each with two yellow spots	***M. careyana***
–	Hypanthium present (0.7–1.4 mm long); petals and sepals upright at anthesis, petals not spotted	***M. scopularum***

## ﻿Discussion

The results of this study indicate that the geographically widespread and morphologically variable *Micranthes virginiensis*, as currently understood, includes populations Gap Creek and Wadakoe Mountain that belong to a morphologically distinct species (to be discussed in detail below). The synonymised taxa that had been recognised by numerous authors throughout the history of this species are confirmed and should remain as such. Both quantitative and qualitative analyses on living and herbarium specimens indicate that the morphological variation within many of the leaf and inflorescence characters used to describe the synonymous taxa are not consistent geographically, but rather occur throughout the range. We have observed that, even within one population, characters like leaf shape, margin type and branching pattern can vary wildly, indicating that these are not reliable characters to use when attempting to delimit species from *M. virginiensis.* Finally, populations in the periphery of the range of *M. virginiensis* (i.e. Arkansas, Maine etc.) do not have consistently distinguishable morphology from populations in the middle of the range.

One pattern throughout the species’ range that we did find is opposition in the direction of the PCA vectors containing leaf and floral characters (Fig. 2a), which appears to indicate a trade-off between leaf size and flower size in this species. This mirrors a trade-off between somatic growth and reproductive allocation that has been observed in other plants (e.g. Thorén et al. (1996); Hemborg and Karlsson (1998); Zhang et al. (2015)). For example, a study of eight subarctic plant species in the Swedish Lapland found a general trade-off between allocation of resources to somatic (including growth and storage) and reproductive functions (Hemborg and Karlsson 1998). Another study observed the flower/leaf size trade-off in *Stellera chamaejasme* populations of the northern Qilian Mountains (Zhang et al. 2015). Hemborg and Karlsson (1998) found that reproductive effort generally decreased with elevation and that reproducing plants incurred somatic costs. They related this to increasing severity of the environment at high altitudes.

Our correlation analysis also found an apparent trade-off in reproductive and somatic size with increasing elevation, although the elevational range of our sampled populations was considerably lower (49.42–727.04 m) than that of the alpine plants in the former studies. Other studies have found a positive correlation between the size of floral and vegetative characters rather than a trade-off (i.e., E-Vojtkó et al. 2022) or changes in reproductive allometry along an elevational gradient, with plants growing at high elevations allocating proportionately more biomass to reproduction at larger sizes and less at smaller sizes than plants growing at lower elevations (Zhang et al. 2020). Therefore, the leaf-flower size trade-off observed in *M. virginiensis* is not observed in all plant species, but also is not unusual.

Some populations currently assigned to *M. virginiensis* that we studied, although not distinct enough based on our data to confidently change species status, may warrant further investigation. The populations of Polk Co., NC. (at Melrose Falls, Pearson’s Falls and Shunkawauken Falls) all appear to share similar floral oddities – the petals are much more spreading, somewhat reflexed in some individuals and the hypanthium is smaller than typical *M. virginiensis*. This is similar to *M. careyana*, though the flowers of the Polk Co. populations do not have petal spots or long stamens. These populations are also on the southern Blue Ridge Escarpment and the localities share a similar geology to the Wadakoe and Gap Creek localities (see below). The Polk Co. plants differ from the Gap Creek and Wadakoe Mountain escarpment populations in having short stamens and yellow anthers, like *M. virginiensis*. Finally, the Polk Co. plants we investigated are diploid (Table 5; Fig. 1).

Though populations we sampled throughout most of the range of *M. virginiensis* were determined to constitute a single species, the Southern Appalachian escarpment populations at Gap Creek and Wadakoe Mountain are a distinct entity under the unified species concept. Since their discovery, these populations were known to differ from typical *M. virginiensis*, though it has been unclear if they represent a new species, a variety, a hybridisation event with a nearby *M. careyana* or simply normal variation within *M. virginiensis.* Using morphological analyses and chromosome counts, we have determined the Gap Creek and Wadakoe Mountain populations to be a distinct species, possibly of hybrid origin between *M. virginiensis* and *M. careyana*.

Other species in *Micranthes* have been determined to be a result of allopolyploidy, such as *M. hitchcockiana* (*n* = 38), a species likely resulting from hybridisation between *M. rufidula* (*n* = 19) and *M. oregana* (*n* = 19) (Elvander 1984). It is not surprising that an undescribed species of hybrid origin should be discovered in the Southern Appalachian escarpment as the prevalence of unique microhabitats has allowed for high rates of biodiversity, endemism and disjunct populations in this region (Pittillo et al. 1998). In particular, this region of north-western South Carolina resides on the Walhalla thrust sheet, much of which contains a mafic suite of rock (amphibolite and amphibole gneiss, fine-grained biotite gneiss and micaceous feldspathic quartzite) underlying relatively circum-neutral soils supporting a rich flora (S. Tessel pers. comm., SCDNR Geological Survey). Wadakoe Mountain, much of which is a South Carolina Department of Natural Resources heritage preserve, is known for its diverse assemblage of uncommon plants due to the underlying amphibolite and circum-neutral pH of the soils (SCDNR (South Carolina Department of Natural Resources)). The Gap Creek site is located at the eastern edge of the Mountain Bridge Wilderness area, managed by the South Carolina Department of Parks, Recreation and Tourism and is mapped as migmatitic granitoid gneiss (Horton and Dicken 2001).

All multivariate analyses (including PCA, LDA and PERMANOVA) indicate that the Gap Creek and Wadakoe Mountain populations have morphology that is intermediate between *M. careyana* and *M. virginiensis*. Specifically, the stamens are more than half the length of the petals in both *M. careyana* and the Gap Creek and Wadakoe Mountain populations, whereas the stamens are less than half the length (and often only around a quarter of the length) of the petals in *M. virginiensis.* Additionally, the hypanthia of the Gap Creek and Wadakoe Mountain populations are generally of intermediate length between the absent hypanthium of *M. careyana* and the distinct hypanthium of *M. virginiensis*, though there is much overlap between the hypanthium lengths of individuals from the Gap Creek and Wadakoe Mountain populations and *M. virginiensis.* The flowers of *M. virginiensis* and the Gap Creek and Wadakoe Mountain populations lack petal spots, while *M. careyana* has yellow/green spots on the flower petals. While the pistil length of the Gap Creek and Wadakoe Mountain populations tended to be greater than the other two species, this trait was not consistent enough to be useful for species delimitation. Fruit size also showed considerable overlap between the three groups and is, thus, also an uninformative taxonomic character. The hybrid origin hypothesis could be further tested through genomic data analyses, which would indicate if these populations contain admixture between alleles from both *M. virginiensis* and *M. careyana*. It could also be tested through artificial crosses that could reveal if *M. virginiensis* and *M. careyana* can hybridise and if those offspring possessed the same unique characters as the Gap Creek and Wadakoe Mountain populations.

Multiple *M. virginiensis* tetraploid populations have been discovered in the southeast beyond the Gap Creek and Wadakoe Mountain populations. These tetraploid populations are not indicated by any of our analyses to be morphologically distinct from diploid *M. virginiensis* and, thus, it is likely that they result from an instance of autotetraploidy or chromosome duplication within a species (Soltis et al. 2007). Based on the continuous distribution of the tetraploids across eastern Virginia, North Carolina and northern South Carolina (Fig. 1), it is possible that a spontaneous autotetraploid population appeared, persisted and spread throughout the region, although autopolyploids are believed to form at a relatively high frequency in natural populations (Ramsey and Schemske 1998) and were shown to have multiple origins in *Galax urceolata* (Servick et al. 2015). Though allopolyploidy, referring to chromosome duplication due to hybridisation, is well known as a catalyst for speciation (Mallet 2007), autopolyploidy has often been considered by taxonomists to represent cytotypes of a single species rather than multiple species due to convention and morphological similarity (Soltis et al. 2007). However, Soltis et al. (2007) argue that autopolyploids can represent distinct evolutionary lineages that can fulfil requirements of multiple species concepts, including biological, taxonomic, diagnosability, apomorphic and evolutionary, despite often exhibiting high morphological similarity to their diploid progenitors (2007). For example, *Galax urceolata* autotetraploids were shown to exhibit a climatic shift relative to their diploid progenitors (Soltis et al. 2007). Soltis et al. (2007) suggested that, in these cases, autopolyploids should be considered separate species to accurately reflect evolution and facilitate conservation, although Soltis (1983) declined to describe the *M. virginiensis* tetraploid populations as such. Further investigations utilising molecular evidence for lineage isolation or a climatic niche divergence analysis might elucidate their status.

Based on clear and consistent morphological differences in the floral morphology, we have determined that the Gap Creek and Wadakoe Mountain populations in the Southern Appalachian escarpment region are worthy of recognition as a distinct species of putative hybrid origin between diploid *M. virginiensis* and *M. careyana*. We have also identified two other potential undescribed species contained within *M. virginiensis*, the diploid Polk County, NC., populations and the presumed autotetraploid populations in NC. and SC., though further study is needed before strong conclusions can be formed regarding the taxonomic status of these groups. After examination of a wealth of living plants and herbarium specimens from all regions where *M. virginiensis* occurs, no evidence was found to support any previously named species, varieties or forms since synonymised with *M. virginiensis*. Future work that could address the lingering questions includes a robust genomic analysis that could confirm any independent lineages or hybridisation, artificial crossing experiments to determine if hybridisation is possible between diploid *M. virginiensis* and *M. careyana*, the Gap Creek and Wadakoe Mountain populations or the autotetraploid populations and morphological analysis of characters not investigated in this study (e.g. seed coat characters, guard cell size) that could reveal cryptic differences between diploid and autotetraploid *M. virginiensis*. The recognition of at least one (and perhaps multiple) distinct species within *M. virginiensis* allows for improved understanding of biodiversity and can inspire others to revisit geographically widespread species to look for other instances where cryptic or morphologically similar species have been overlooked.

## ﻿Conclusions

This study reveals an undescribed, narrowly endemic species of possibly hybrid origin from the Southern Blue Ridge Escarpment region, *Micranthes scopularum*, based on qualitative and quantitative morphological traits and chromosome number. In addition, it provides a formal description and key to identification of the new species. We present the first karyotypic data for the south-eastern U.S. *Micranthes* species, *M. scopularum*, *M. careyana*, *M. palmeri* and *M. petiolaris*, as well as for multiple populations of *M. virginiensis* throughout its range and confirm previously documented cases of polyploid populations. This is done within the context of summarising the synonymy and morphological and cytological variation within the widespread *M. virginiensis*. Our findings indicate that future studies are warranted to better understand the causes and consequences of polyploidy in this taxon.

## Supplementary Material

XML Treatment for Micranthes scopularum

## ﻿Additional information

### Use of AI

No use of AI was reported.

### Author ORCIDs

Luiz F. L. da Silveira https://orcid.org/0000-0002-0648-3993

Katherine G. Mathews https://orcid.org/0000-0002-9051-3453

### Data availability

All of the data that support the findings of this study are available in the main text or Supplementary Information.

## Supplementary materials

Supplementary material 1 Supplementary figure and tables Data type docx https://binary.pensoft.net/file/1491246This dataset is made available under the Open Database License (http://opendatacommons.org/licenses/odbl/1.0/). The Open Database License (ODbL) is a license agreement intended to allow users to freely share, modify, and use this Dataset while maintaining this same freedom for others, provided that the original source and author(s) are credited.Tara K. Hall, Luiz F. L. da Silveira, Max S. Lanning, Katherine G. Mathews
