# Supplementary material for: ﻿Taxonomy, morphology and cytology of Micranthes virginiensis (Michaux) Small (Saxifragales, Saxifragaceae), new chromosome counts for Micranthes and description of a new Micranthes species from the south-eastern USA
Source: PhytoKeys. 2025 Dec 12;267:309–43. doi: 10.3897/phytokeys.267.162132 (PMC12717515; doi:10.3897/phytokeys.267.162132)
Supplement: Supplementary material 1 — Supplementary figure and tables [file phytokeys-267-309_article-162132__-s001.docx]

APPENDIX: SUPPLEMENTARY FIGURES AND TABLES

Supplementary Figure 1. Biplots of log geographical variables (latitude, longitude, and elevation) as predictors of size (represented by PCA1 scores; A, C, E) or somatic-reproductive allocation (represented by PCA2 scores; B, D, F) in *M*. *virginiensis*. The only plots with statistically significant relationships are B and D, showing somatic trait sizes increasing and reproductive trait sizes decreasing with elevation and longitude Slopes and standard error of the slope estimated by ordinary least squares regression shown on B and D.

Supplementary Table 1: Voucher specimens. All specimens are deposited at WCUH.

| Species | Year | Coll. Date | Country | State | County | Latitude | Longitude | Chrom. # | Hall Coll. # | Permit # |
| --- | --- | --- | --- | --- | --- | --- | --- | --- | --- | --- |
| careyana | 2022 | 1-Apr | USA | NC | Swain | 35.33403 | -83.624428 |  | 13 |  |
| careyana | 2023 | 16-Apr | USA | NC | McDowell | 35.700737 | -82.19558 | 10 | 53 |  |
| careyana | 2022 | 23-Apr | USA | NC | Macon | 35.117584 | -83.270458 | 10 | 31 |  |
| careyana | 2022 | 23-Apr | USA | NC | McDowell | 35.700737 | -82.19558 |  | 32 | File Code: 2720 |
| careyana | 2022 | 10-Apr | USA | TN | Knox | 35.955775 | -83.863 | 10 | 20 |  |
| micranthidifolia | 2022 | 11-Apr | USA | NC | Jackson | 35.345428 | -83.164555 | 12 | 15 |  |
| palmeri | 2022 | 6-Apr | USA | AR | Conway | 35.288696 | -92.483929 | 10 | 8 |  |
| palmeri | 2022 | 1-Apr | USA | MO | Douglas | 36.82929 | -92.4244 | 10 | 16 | No # |
| petiolaris | 2022 | 23-Apr | USA | NC | Macon | 35.117584 | -83.270458 | 10-13 | 30 |  |
| petiolaris | 2023 | 11-Jun | USA | NC | Ashe | 36.406256 | -81.467033 | 10 | 47 |  |
| virginiensis | 2022 | 21-Mar | USA | AL | Jefferson | 33.703495 | -86.692383 | 10 | 9 |  |
| virginiensis | 2022 | 28-Mar | USA | AR | Pulaski | 34.801828 | -92.32132 | 10 | 6 |  |
| virginiensis | 2022 | 11-May | USA | CT | New Haven | 41.55783 | -72.759383 | 10 | 41 |  |
| virginiensis | 2022 | 30-Mar | USA | GA | Fulton | 33.882412 | -84.440262 | 10 | 10 |  |
| virginiensis | 2022 | 13-Apr | USA | KY | Clinton | 36.871587 | -85.192345 |  | 23 |  |
| virginiensis | 2022 | 13-Apr | USA | KY | Todd | 36.921097 | -87.285705 | 10 | 22 |  |
| virginiensis | 2022 | 14-Apr | USA | KY | Franklin | 38.218933 | -84.847183 |  | 25 |  |
| virginiensis | 2022 | 11-May | USA | MA | Middlesex | 42.278647 | -71.343405 |  | 40 |  |
| virginiensis | 2022 | 10-May | USA | MD | Montgomery | 39.088158 | -77.124992 | 10 | 38 | No # |
| virginiensis | 2022 | 12-May | USA | ME | Knox | 44.254487 | -69.095772 |  | 42 |  |
| virginiensis | 2022 | 18-May | USA | MI | Marquette | 46.761716 | -87.73377 | 10 | 39 |  |
| virginiensis | 2022 | 18-May | USA | MI | Marquette | 46.854202 | -87.859005 |  | 43 |  |
| virginiensis | 2022 | 11-Mar | USA | MS | Clay | 33.537987 | -88.633453 | 10 | 11 |  |
| virginiensis | 2022 | 14-Mar | USA | NC | Polk | 35.221517 | -82.305908 | 10 | 1 |  |
| virginiensis | 2023 | 16-Mar | USA | NC | Mecklenburg | 35.152347 | -80.736725 | 19 | 50 |  |
| virginiensis | 2022 | 27-Mar | USA | NC | Polk | 35.272053 | -82.216317 | 10 | 2 |  |
| virginiensis | 2022 | 27-Mar | USA | NC | Durham | 36.072998 | -78.864864 | 19 | 5 |  |
| virginiensis | 2022 | 6-Apr | USA | NC | Polk | 35.272053 | -82.216317 | 10 | 46 |  |
| virginiensis | 2022 | 4-May | USA | NC | Chatham | 35.736786 | -79.112814 | 19 | 34 |  |
| virginiensis | 2023 | 16-Mar | USA | NC | Montgomery | 35.40607 | -80.09268 | 19 | 51 |  |
| virginiensis | 2022 | 28-Mar | USA | NJ | Somerset | 40.584764 | -74.559456 | 10 | 12 | via email |
| virginiensis | 2022 | 27-Apr | USA | NY | Tompkins | 42.399242 | -76.53585 |  | 27 | via email |
| virginiensis | 2022 | 14-May | USA | NY | Erie | 42.700905 | -78.904725 |  | 45 |  |
| virginiensis | 2022 | 14-Apr | USA | OH | Hamilton | 39.124675 | -84.782745 |  | 26 |  |
| virginiensis | 2022 | 15-Apr | USA | OH | Fairfield | 39.63158 | -82.647383 |  | 29 |  |
| virginiensis | 2022 | 10-May | USA | PA | Chester | 39.727925 | -76.073142 |  | 37 | 22-827 |
| virginiensis | 2022 | 14-Mar | USA | SC | Pickens | 34.9005288 | -82.6593307 | 22 | 4 |  |
| virginiensis | 2022 | 6-Apr | USA | SC | Spartanburg | 35.140617 | -82.278862 | 10 | 19 |  |
| virginiensis | 2022 | 10-Apr | USA | TN | Davidson | 36.053741 | -86.91092 | 11 | 21 |  |
| virginiensis | 2022 | 13-Apr | USA | TN | Jefferson | 36.101922 | -83.627633 |  | 24 |  |
| virginiensis | 2022 | 21-Mar | USA | VA | Powhatan | 37.682778 | -77.938333 | 10 | 7 | PW-RCP-020822 |
| virginiensis | 2022 | 9-May | USA | VA | Floyd | 36.803506 | -80.341778 |  | 36 | BLRI-2022-SCI-0017 |
| virginiensis | 2022 | 27-Apr | USA | VT | Orange | 43.919051 | -72.210572 |  | 18 |  |
| virginiensis | 2022 | 15-Apr | USA | WV | Wayne | 38.146458 | -82.382308 |  | 28 |  |
| virginiensis | 2022 | 18-May | CAN | ONT | Lennox | 44.53755 | -76.92789 |  | 33 |  |
| virginiensis | 2022 | 18-May | CAN | ONT | Lennox | 44.560672 | -77.116398 | 10 | 35 |  |
| virginiensis | 2022 | 13-May | CAN | QBC | Le Haut-Richelieu | 45.354428 | -73.150506 |  | 44 |  |
| virginiensis  (Gap Creek) | 2022 | 14-Mar | USA | SC | Greenville | 35.164122 | -82.475519 | 19 | 3 | N-2-23 |
| virginiensis  (Wadakoe Mtn.) | 2022 | 26-Mar | USA | SC | Pickens | 34.98221 | -82.84356 | 19 | 14 | SC-92-2022 |

Supplementary Table 2. *M. virginiensis* specimens used for PCA conducted with fruit characters.

| Specimen ID | Year | Date | State | County | Latitude | Longitude |
| --- | --- | --- | --- | --- | --- | --- |
| UNA00034416 | 1982 | 16-Apr | AL | Wilcox | 31.908333 | -87.380556 |
| UNA00034491 | 1982 | 9-Apr | AL | Dallas | 32.32 | -83.03 |
| UNA00034496 | 1982 | 7-Apr | AL | Lowndes | 32.353611 | -86.690833 |
| NCU00090765 | 1967 | 16-Apr | AL | Randolph | 33.279788 | -85.645296 |
| UNA00014924 | 1977 | 2-Apr | AL | Walker | 33.614444 | -87.363333 |
| UNA00065282 | 2003 | 9-Apr | AL | Lawrence | 34.395833 | -87.215278 |
| NCU00090772 | 1967 | 30-Apr | AR | Franklin | 35.673382 | -93.699275 |
| NCU00090770 | 1967 | 31-Mar | AR | Cleburne | 35.459218 | -92.03591 |
| ANHC009866 | 2016 | 9-Apr | AR | Pulaski | 34.80187 | -92.32307 |
| UVMVT068793 | 1974 | 19-May | CT | Hartford | 41.65711 | -72.66329 |
| NCU00090930 | 1897 | 7-May | DC | Washington, D.C. | 38.895112 | -77.036366 |
| PH00498081 | 1881 | 10-May | DE | New Castle | 39.739001 | -75.635761 |
| NCU00090787 | 1964 | 23-Apr | GA | Walton | 33.765827 | -83.852404 |
| CLEMS0066960 | 1978 | 4-Apr | GA | Elbert | 34.2575 | -82.747778 |
| GA035914 | 1986 | 21-Apr | GA | Cherokee | 34.317418 | -84.645479 |
| NCU00090982 | 1949 | 26-Apr | IL | Hardin | 37.560192 | -88.120932 |
| IND-0046733 | 1927 | 24-Apr | IL | Crawford | 38.182216 | -86.381489 |
| IND-0046741 | 1929 | 5-May | IN | Spencer | 37.886783 | -87.046321 |
| IND-0046743 | 1941 | 15-Apr | KY | Warren | 37.083509 | -86.579398 |
| NCU00090791 | 1963 | 5-May | KY | Henry | 38.364896 | -84.880879 |
| MARY1018304 | 1966 | 15-May | MD | Baltimore | 39.443768 | -76.510269 |
| DOV0036330 | 1997 | 17-May | MD | Allegany | 39.665667 | -78.462833 |
| NCU00090907 | 1969 | 16-May | MD | Washington | 39.637175 | -78.329943 |
| MARY1018308 | 1980 | 17-May | MD | Allegany | 39.693337 | -78.451993 |
| HUDC00009878 | 1967 | 27-May | MD | Allegany | 39.636887 | -78.457557 |
| MARY1018360 | 1947 | 11-May | MD | Montgomery | 39.152383 | -77.120321 |
| UVMVT144783 | 1984 | 10-May | MD | Prince Georges | 38.473681 | -77.013484 |
| 4737 | 1914 | 30-May | MA | Worcester | 42.5834 | -71.8023 |
| IND-0046746 | 1905 | 28-May | MA | Middlesex | 42.345801 | -71.450001 |
| 1465255 | 1970 | 16-Jun | MI | Keweenaw | 48.099098 | -88.601638 |
| 1465257 | 1930 | 30-Jun | MI | Keweenaw | 48.12023 | -88.53492 |
| 1477443 | 1958 | 30-May | MI | Ontonagon | 46.693192 | -89.732307 |
| 1465249 | 1957 | 6-Jul | MI | Keweenaw | 48.044027 | -88.701576 |
| 1465270 | 1958 | 30-May | MI | Ontonagon | 46.76667 | -89.75 |
| 1465253 | 1979 | 18-Jun | MI | Chippewa | 46.075773 | -83.666114 |
| UNCC_45631 | 1984 | 10-Apr | MS | Tishomingo | 34.6024 | -88.1938 |
| MMNS006411 | 1979 | 9-Apr | MS | Tishomingo | 34.93403 | -88.17902 |
| 59163 | 1963 | 20-Apr | MO | Douglas | 39.4 | -93.8167 |
| ANHC010841 | 2009 | 22-Apr | MO | Shannon | 37.11615 | -91.19997 |
| NCU00090991 | 1887 | 14-Apr | MO | Jefferson | 38.261071 | -90.537689 |
| UVMVT068805 | 1969 | 12-Jun | NH | Strafford | 43.44935 | -71.00751 |
| PH00498301 | 1936 | 24-May | NJ | Cape May | 38.987613 | -74.95323 |
| PH00498241 | 1922 | 22-May | NJ | Monmouth | 40.106692 | -74.518673 |
| 1246908 | 1892 | 1-May | NY | Bronx | 40.856767 | -73.875414 |
| SIM0003763 | 1885 | 21-Jun | NY | Richmond | 40.625278 | -74.095833 |
| NCU00088179 | 1957 | 25-Apr | NC | Vance | 36.324846 | -78.375974 |
| NCU00088161 | 1958 | 6-Apr | NC | Lee | 35.575831 | -79.201701 |
| NCU00088181 | 1938 | 11-Apr | NC | Wake | 35.830113 | -78.638615 |
| NCU00088142 | 1958 | 22-May | NC | Caswell | 36.28707 | -79.221237 |
| NCU00088173 | 1992 | 24-Apr | NC | Surrey | 36.55013 | -80.908687 |
| NCU00088176 | 1974 | 21-Apr | NC | Stokes | 36.429051 | -80.298403 |
| NCU00088173 | 1992 | 24-Apr | NC | Surrey | 36.55013 | -80.908687 |
| NCU00088177 | 1958 | 4-May | NC | Stokes | 36.429951 | -80.288942 |
| NCU00090960 | 1959 | 10-Jul | ONT | Thunder Bay | 48.751251 | -87.975253 |
| PH00498115 | 1947 | 5-Jun | PA | Wayne | 41.610825 | -75.060752 |
| PH00497956 | 1923 | 30-May | PA | Bucks | 40.387142 | -75.181386 |
| PH00498196 | 1957 | 23-May | PA | Clearfield | 41.069224 | -78.367683 |
| PH00498109 | 1937 | 17-May | PA | Butler | 40.855111 | -80.098514 |
| PH00498199 | 1946 | 5-Jun | PA | Indiana | 40.869762 | -79.094215 |
| PH00498182 | 1946 | 11-May | PA | Franklin | 40.151347 | -77.715536 |
| PH00497914 | 1921 | 8-May | PA | Lehigh | 40.560523 | -75.572792 |
| IND-0046755 | 1917 | 1-Jun | QBC | Cap-a-la-Branche | 47.384136 | -70.429108 |
| PBRU00056700 | 2016 | 12-May | RI | Providence | 41.91861 | -71.44625 |
| CLEMS0067009 | 1992 | 20-Apr | SC | Newberry | 34.497412 | -81.58919 |
| USCH0057789 | 2012 | 11-Apr | SC | McCormick | 33.6863 | -82.1697 |
| CLEMS0067011 | 1987 | 25-Apr | SC | Oconee | 34.757904 | -83.197198 |
| CLEMS0067017 | 1974 | 22-Mar | SC | Richland | 34.096051 | -81.126246 |
| CLEMS0067014 | 2002 | 19-Apr | SC | Pickens | 34.980175 | -82.843122 |
| WCUH0024319 | 2008 | 19-Apr | SC | Pickens | 34.98221 | -82.84356 |
| CLEMS0067008 | 1978 | 9-Apr | SC | Laurens | 34.498504 | -82.139386 |
| CLEMS0067012 | 1986 | 26-Apr | SC | Oconee | 34.757904 | -83.197198 |
| NCU00090720 | 1957 | 14-Apr | SC | York | 34.904823 | -81.461335 |
| APSC0003160 | 2010 | 15-Apr | TN | Jackson | 36.4239 | -85.6497 |
| NCU00090910 | 1935 | 24-Mar | TN | Davidson | 36.16589 | -86.784443 |
| WCUH0024320 | 1973 | 12-Apr | TN | Smith | 36.14249 | -85.823105 |
| UVMVT068755 | 1908 | 1-Jun | VT | Bennington | 43.25879 | -73.05147 |
| UVMVT068741 | 1959 | 30-May | VT | Chittenden | 44.53826 | -72.88613 |
| UVMVT068723 | 1892 | 15-May | VT | Chittenden | 44.48735 | -73.23124 |
| UVMVT068708 | 1967 | 13-May | VT | Bennington | 42.79188 | -73.21203 |
| 18702 | 1975 | 2-May | VA | New Kent | 37.485776 | -76.784858 |
| NCU00092555 | 1966 | 7-May | VA | Rockingham | 38.302073 | -78.622517 |
| 1400883 | 2011 | 10-May | VA | Patrick | 36.606569 | -80.449547 |
| WVA-V-0068747 | 1984 | 27-Apr | WV | Summers | 37.587332 | -80.745956 |
| WVA-V-0025989 | 2014 | 17-May | WV | Calhoun | 38.828533 | -81.147217 |
| WVA-V-0068718 | 1952 | 10-May | WV | Pocahontas | 38.90027778 | -78.15916667 |
| WVA-V-0068668 | 1985 | 24-Apr | WV | Fayette | 38.15 | -81.2 |
| WVA-V-0068728 | 1998 | 3-Jun | WV | Pendleton | 38.826779 | -79.29143 |
| WVA-V-0068706 | 2001 | 26-Apr | WV | Monongalia | 39.55 | -80 |
| WVA-V-0068654 | 1891 | 1-Apr | WV | Fayette | 37.97161 | -81.154165 |
| WVA-V-0068674 | 1939 | 6-May | WV | Jefferson | 39.492599 | -77.780272 |
| WVA-V-0068669 | 2008 | 2-May | WV | Boone | 38.155429 | -81.644985 |

Supplementary Table 3. All *M. virginiensis* specimens imaged in SERNEC (Southeast Regional Network of Expertise and Collections online database) examined in this study.

| Specimen ID | Year | Date | State/Province | County | Latitude | Longitude |
| --- | --- | --- | --- | --- | --- | --- |
| TROY000042226 | 2012 | 15-Mar | AL | Butler | 31.916959 | -86.688774 |
| UNA00034491 | 1982 | 9-Apr | AL | Dallas | 32.32 | -83.03 |
| TENN-V-0229552 | 1993 | 5-Apr | AL | Jefferson | 33.772864 | -86.841349 |
| UNA00065282 | 2003 | 9-Apr | AL | Lawrence | 34.395833 | -87.215278 |
| UNA00034496 | 1982 | 7-Apr | AL | Lowndes | 32.353611 | -86.690833 |
| UNA00014923 | 1979 | 15-Mar | AL | Marshall | 34.41 | -86.39 |
| NCU00090765 | 1967 | 16-Apr | AL | Randolph | 33.279788 | -85.645296 |
| UNA00014924 | 1977 | 2-Apr | AL | Walker | 33.614444 | -87.363333 |
| UNA00034416 | 1982 | 16-Apr | AL | Wilcox | 31.908333 | -87.380556 |
| UNA00065427 | 2005 | 19-Apr | AL | Winston | 34.09 | -87.61 |
| NCU00090770 | 1967 | 31-Mar | AR | Cleburne | 35.459218 | -92.03591 |
| ANHC007463 | 2006 | 28-Mar | AR | Drew | 33.73613 | -91.62441 |
| NCU00090772 | 1967 | 30-Apr | AR | Franklin | 35.673382 | -93.699275 |
| 276702 | 2016 | 6-Apr | AR | Pulaski | 34.8018 | -92.3213 |
| ANHC009866 | 2016 | 9-Apr | AR | Pulaski | 34.80187 | -92.32307 |
| UVMVT068793 | 1974 | 19-May | CT | Hartford | 41.65711 | -72.66329 |
| NCU00090930 | 1897 | 7-May | DC | Washington, D.C. | 38.895112 | -77.036366 |
| PH00498083 | 1897 | 29-Apr | DE | New Castle | 39.788368 | -75.636863 |
| PH00498081 | 1881 | 10-May | DE | New Castle | 39.739001 | -75.635761 |
| GA035914 | 1986 | 21-Apr | GA | Cherokee | 34.317418 | -84.645479 |
| WCUH0024322 | 2006 | 16-May | GA | Cobb | 33.953698 | -84.592143 |
| CLEMS0066961 | 1978 | 4-Apr | GA | Elbert | 34.052856 | -82.645015 |
| CLEMS0066960 | 1978 | 4-Apr | GA | Elbert | 34.2575 | -82.747778 |
| NCU00090787 | 1964 | 23-Apr | GA | Walton | 33.765827 | -83.852404 |
| IND-0046733 | 1927 | 24-Apr | IL | Crawford | 38.182216 | -86.381489 |
| NCU00090982 | 1949 | 26-Apr | IL | Hardin | 37.560192 | -88.120932 |
| IND-0046734 | 1934 | 29-Apr | IN | Dearborn | 38.987077 | -85.022697 |
| IND-0046741 | 1929 | 5-May | IN | Spencer | 37.886783 | -87.046321 |
| NCU00090789 | 1955 | 6-Apr | KY | Fayette | 37.902746 | -84.397728 |
| MUHW031902 | 1937 | 14-May | KY | Hancock | 37.896533 | -86.755675 |
| NCU00090791 | 1963 | 5-May | KY | Henry | 38.364896 | -84.880879 |
| HTTU034555 | 1998 | 28-Mar | KY | Metcalfe | 36.977 | -85.696167 |
| IND-0046743 | 1941 | 15-Apr | KY | Warren | 37.083509 | -86.579398 |
| NCU00090798 | 1959 | 6-Mar | LA | Union | 32.729938 | -92.405699 |
| IND-0046746 | 1905 | 28-May | MA | Middlesex | 42.345801 | -71.450001 |
| 4737 | 1914 | 30-May | MA | Worcester | 42.5834 | -71.8023 |
| DOV0036330 | 1997 | 17-May | MD | Allegany | 39.665667 | -78.462833 |
| MARY1018308 | 1980 | 17-May | MD | Allegany | 39.693337 | -78.451993 |
| HUDC00009878 | 1967 | 27-May | MD | Allegany | 39.636887 | -78.457557 |
| MARY1018304 | 1966 | 15-May | MD | Baltimore | 39.443768 | -76.510269 |
| MARY1018360 | 1947 | 11-May | MD | Montgomery | 39.152383 | -77.120321 |
| UVMVT144783 | 1984 | 10-May | MD | Prince Georges | 38.473681 | -77.013484 |
| NCU00090907 | 1969 | 16-May | MD | Washington | 39.637175 | -78.329943 |
| UVMVT068804 | 1999 | 18-May | ME | Androscoggin | 44.09146 | -70.16808 |
| 1465248 | 1985 | 26-May | MI | Chippewa | 46.07868 | -83.644765 |
| 1465253 | 1979 | 18-Jun | MI | Chippewa | 46.075773 | -83.666114 |
| 1465255 | 1970 | 16-Jun | MI | Keweenaw | 48.099098 | -88.601638 |
| 1465257 | 1930 | 30-Jun | MI | Keweenaw | 48.12023 | -88.53492 |
| 1465249 | 1957 | 6-Jul | MI | Keweenaw | 48.044027 | -88.701576 |
| 1477443 | 1958 | 30-May | MI | Ontonagon | 46.693192 | -89.732307 |
| 1465270 | 1958 | 30-May | MI | Ontonagon | 46.76667 | -89.75 |
| 906510 | 2008 | 18-Jun | MN | Cook | 47.895 | -90.56 |
| 178406 | 1894 | 10-Aug | MN | Lake of the Woods | 49.353711 | -95.002845 |
| 928696 | 2010 | 10-Jun | MN | Saint Louis | 47.8002778 | -92.0622222 |
| 59163 | 1963 | 20-Apr | MO | Douglas | 39.4 | -93.8167 |
| NCU00090991 | 1887 | 14-Apr | MO | Jefferson | 38.261071 | -90.537689 |
| ANHC010841 | 2009 | 22-Apr | MO | Shannon | 37.11615 | -91.19997 |
| UNCC_45631 | 1984 | 10-Apr | MS | Tishomingo | 34.6024 | -88.1938 |
| MMNS006411 | 1979 | 9-Apr | MS | Tishomingo | 34.93403 | -88.17902 |
| NCU00088139 | 1958 | 2-May | NC | Alleghany | 36.571007 | -81.2 |
| NCU00088142 | 1958 | 22-May | NC | Caswell | 36.28707 | -79.221237 |
| NCU00088145 | 1960 | 18-Apr | NC | Catawba | 35.604469 | -80.943845 |
| NCU00088147 | 1956 | 22-Mar | NC | Cleveland | 35.201363 | -81.665131 |
| NCU00088150 | 1958 | 23-Apr | NC | Edgecombe | 35.959087 | -77.781101 |
| NCU00088152 | 1958 | 17-May | NC | Forsyth | 36.183674 | -80.073653 |
| NCU00088156 | 1956 | 26-Apr | NC | Granville | 36.194807 | -78.582936 |
| NCU00088162 | 1958 | 15-Apr | NC | Lee | 35.580857 | -79.154666 |
| NCU00088161 | 1958 | 6-Apr | NC | Lee | 35.575831 | -79.201701 |
| CLEMS0066966 | 1958 | 18-Apr | NC | Mecklenburg | 35.500153 | -80.832807 |
| NCU00088176 | 1974 | 21-Apr | NC | Stokes | 36.429051 | -80.298403 |
| NCU00088177 | 1958 | 4-May | NC | Stokes | 36.429951 | -80.288942 |
| NCU00088173 | 1992 | 24-Apr | NC | Surrey | 36.55013 | -80.908687 |
| NCU00088178 | 1956 | 16-Apr | NC | Surry | 36.277438 | -80.770647 |
| NCU00088179 | 1957 | 25-Apr | NC | Vance | 36.324846 | -78.375974 |
| NCU00088181 | 1938 | 11-Apr | NC | Wake | 35.830113 | -78.638615 |
| UVMVT068805 | 1969 | 12-Jun | NH | Strafford | 43.44935 | -71.00751 |
| CM453972 | 1923 | 28-Apr | NJ | Burlington | 39.912305 | -74.810137 |
| PH00498301 | 1936 | 24-May | NJ | Cape May | 38.987613 | -74.95323 |
| PH00498241 | 1922 | 22-May | NJ | Monmouth | 40.106692 | -74.518673 |
| PH00498313 | 1938 | 1-May | NJ | Somerset | 40.448585 | -74.756494 |
| PAC0042447 | 1927 | 16-May | NY | Albany | 42.604802 | -73.769566 |
| 1246908 | 1892 | 1-May | NY | Bronx | 40.856767 | -73.875414 |
| KHD00051421 | 1939 | 11-May | NY | Monroe | 43.173105 | -77.709017 |
| SIM0003761 | 1881 | 1-May | NY | Richmond | 40.635638 | -74.092162 |
| SIM0003763 | 1885 | 21-Jun | NY | Richmond | 40.625278 | -74.095833 |
| CM396722 | 1993 | 7-May | NY | Ulster | 41.989476 | -74.244577 |
| NCU00090960 | 1959 | 10-Jul | ON | Thunder Bay | 48.751251 | -87.975253 |
| CM051049 | 1951 | 12-May | PA | Armstrong | 40.877908 | -79.440748 |
| CM537111 | 2016 | 21-May | PA | Bedford | 39.81707 | -78.40278 |
| MOAR0015267 | 2005 | 5-Apr | PA | Bucks | 40.515294 | -75.09192 |
| PH00497956 | 1923 | 30-May | PA | Bucks | 40.387142 | -75.181386 |
| PH00498109 | 1937 | 17-May | PA | Butler | 40.855111 | -80.098514 |
| CM051032 | 1924 | 1-May | PA | Chester | 39.895387 | -75.734384 |
| PH00498196 | 1957 | 23-May | PA | Clearfield | 41.069224 | -78.367683 |
| CM051038 | 1970 | 6-May | PA | Clinton | 41.277475 | -77.885329 |
| CM469925 | 2005 | 8-Jun | PA | Erie | 42.01745 | -80.390603 |
| PH00498182 | 1946 | 11-May | PA | Franklin | 40.151347 | -77.715536 |
| CM495302 | 1996 | 10-May | PA | Fulton | 39.73333 | -78.33333 |
| CM050960 | 1952 | 13-May | PA | Huntingdon | 40.228339 | -78.050899 |
| PH00498199 | 1946 | 5-Jun | PA | Indiana | 40.869762 | -79.094215 |
| PH00498076 | 1960 | 2-May | PA | Lancaster | 40.178826 | -76.082168 |
| CM050986 | 1952 | 3-May | PA | Lawrence | 40.856203 | -80.315999 |
| PH00497914 | 1921 | 8-May | PA | Lehigh | 40.560523 | -75.572792 |
| PH00498136 | 1937 | 22-May | PA | Schuylkill | 40.641123 | -76.600521 |
| PH00498115 | 1947 | 5-Jun | PA | Wayne | 41.610825 | -75.060752 |
| IND-0046755 | 1917 | 1-Jun | QB | Cap-a-la-Branche | 47.384136 | -70.429108 |
| 129120 | 1957 | 23-May | QB | Charlevoix | 47.4384 | -70.4631 |
| PBRU00056700 | 2016 | 12-May | RI | Providence | 41.91861 | -71.44625 |
| ASU0131689 | 1957 | 12-Apr | SC | Laurens | 34.454803 | -82.198702 |
| CLEMS0067008 | 1978 | 9-Apr | SC | Laurens | 34.498504 | -82.139386 |
| USCH0057789 | 2012 | 11-Apr | SC | McCormick | 33.6863 | -82.1697 |
| CLEMS0067009 | 1992 | 20-Apr | SC | Newberry | 34.497412 | -81.58919 |
| CLEMS0067011 | 1987 | 25-Apr | SC | Oconee | 34.757904 | -83.197198 |
| CLEMS0067012 | 1986 | 26-Apr | SC | Oconee | 34.757904 | -83.197198 |
| CLEMS0067016 | 1974 | 22-Mar | SC | Richland | 34.099859 | -81.111838 |
| CLEMS0067017 | 1974 | 22-Mar | SC | Richland | 34.096051 | -81.126246 |
| NCU00090724 | 1975 | 6-Apr | SC | York | 34.8625 | -81.09492 |
| NCU00090720 | 1957 | 14-Apr | SC | York | 34.904823 | -81.461335 |
| NCU00090913 | 1961 | 18-Apr | TN | Cheatham | 36.246058 | -87.017755 |
| 276703 | 2016 | 12-Apr | TN | Coffee | 35.4856 | -86.1062 |
| NCU00090914 | 1964 | 14-Apr | TN | Davidson | 36.095409 | -86.533971 |
| NCU00090910 | 1935 | 24-Mar | TN | Davidson | 36.16589 | -86.784443 |
| HTTU016081 | 1999 | 16-Apr | TN | Dekalb | 36 | -85.666 |
| APSC0003160 | 2010 | 15-Apr | TN | Jackson | 36.4239 | -85.6497 |
| GA155493 | 1949 | 31-Mar | TN | Polk | 35.219754 | -84.519161 |
| WCUH0024320 | 1973 | 12-Apr | TN | Smith | 36.14249 | -85.823105 |
| NCU00092526 | 1975 | 24-Apr | VA | Carroll | 36.892739 | -80.712262 |
| 57763 | 1991 | 4-May | VA | Greene | 38.378211 | -78.511065 |
| WVA-V-0016587 | 1964 | 11-Apr | VA | James City | 37.145595 | -76.733115 |
| GMUF-0042091 | 2017 | 2-Apr | VA | Loudoun | 39.2895 | -77.737189 |
| 18702 | 1975 | 2-May | VA | New Kent | 37.485776 | -76.784858 |
| 1400883 | 2011 | 10-May | VA | Patrick | 36.606569 | -80.449547 |
| NCU00092553 | 1966 | 30-Apr | VA | Prince Edward | 37.25815 | -78.414276 |
| NCU00092555 | 1966 | 7-May | VA | Rockingham | 38.302073 | -78.622517 |
| ODU00024347 | 1992 | 24-Apr | VA | Surry | 36.55013 | -80.908687 |
| Benn-2188 | 1975 | 19-May | VT | Bennington | 42.793086 | -73.255313 |
| UVMVT068755 | 1908 | 1-Jun | VT | Bennington | 43.25879 | -73.05147 |
| UVMVT068708 | 1967 | 13-May | VT | Bennington | 42.79188 | -73.21203 |
| UVMVT068741 | 1959 | 30-May | VT | Chittenden | 44.53826 | -72.88613 |
| UVMVT068723 | 1892 | 15-May | VT | Chittenden | 44.48735 | -73.23124 |
| UVMVT068751 | 1977 | 1-May | VT | Westmore | 44.76016 | -72.02697 |
| UVMVT068717 | 1937 | 16-May | VT | Williston | 44.43457 | -73.08868 |
| WVA-V-0068669 | 2008 | 2-May | WV | Boone | 38.155429 | -81.644985 |
| WVA-V-0025989 | 2014 | 17-May | WV | Calhoun | 38.828533 | -81.147217 |
| WVA-V-0068668 | 1985 | 24-Apr | WV | Fayette | 38.15 | -81.2 |
| WVA-V-0068654 | 1891 | 1-Apr | WV | Fayette | 37.97161 | -81.154165 |
| NCU00090921 | 1970 | 4-May | WV | Hampshire | 39.222223 | -78.845537 |
| WVA-V-0068674 | 1939 | 6-May | WV | Jefferson | 39.492599 | -77.780272 |
| WVA-V-0068712 | 2002 | 16-Apr | WV | Mason | 38.685413 | -82.034138 |
| WVA-V-0068710 | 2002 | 10-Apr | WV | McDowell | 37.4566 | -81.882144 |
| WVA-V-0068706 | 2001 | 26-Apr | WV | Monongalia | 39.55 | -80 |
| WVA-V-0068728 | 1998 | 3-Jun | WV | Pendleton | 38.826779 | -79.29143 |
| WVA-V-0068734 | 2013 | 17-Apr | WV | Pleasants | 39.41505 | -81.08415 |
| WVA-V-0068718 | 1952 | 10-May | WV | Pocahontas | 38.90027778 | -78.15916667 |
| WVA-V-0068747 | 1984 | 27-Apr | WV | Summers | 37.587332 | -80.745956 |

Supplementary Table 4. Leaf and floral measurements used in multivariate analyses. “escarpment” = Gap Creek and Wadakoe Mountain.

| Species | | Hypanthium Length (mm) | Stamen Length (mm) | | Petal Length (mm) | Petal Width (mm) | | Pistil Length (mm) | | Plant Height (cm) | |
| --- | --- | --- | --- | --- | --- | --- | --- | --- | --- | --- | --- |
| virginiensis | | 1.668 | 1.574 | | 6.356 | 2.316 | | 3.782 | | 24 | |
| virginiensis | | 0.777 | 1.279 | | 4.873 | 1.638 | | 1.243 | | 16.2 | |
| virginiensis | | 1.749 | 1.802 | | 6.654 | 1.937 | | 4.036 | | 21.4 | |
| virginiensis | | 0.87 | 1.49 | | 5.3 | 2.13 | | 2.251 | | 16.1 | |
| virginiensis | | 1.145 | 1.532 | | 5.392 | 2.11 | | 2.422 | | 9 | |
| virginiensis | | 1.1 | 1.37 | | 4.657 | 2.312 | | 2.068 | | 21.4 | |
| virginiensis | | 1.375 | 1.417 | | 4.648 | 1.625 | | 2.292 | | 21.9 | |
| virginiensis | | 0.603 | 0.908 | | 2.876 | 1.57 | | 2.612 | | 7.4 | |
| virginiensis | | 1.012 | 1.466 | | 4.619 | 1.8 | | 2.275 | | 14.5 | |
| virginiensis | | 1.211 | 1.449 | | 4.467 | 1.953 | | 3.059 | | 13.2 | |
| virginiensis | | 1.112 | 1.517 | | 4.499 | 1.471 | | 1.634 | | 16.1 | |
| virginiensis | | 0.91 | 1.12 | | 3.21 | 1.32 | | 2.218 | | 16.5 | |
| virginiensis | | 1.707 | 1.841 | | 5.12 | 2.511 | | 1.904 | | 12.3 | |
| virginiensis | | 1.189 | 1.731 | | 4.724 | 2.147 | | 1.944 | | 16.8 | |
| virginiensis | | 1.454 | 1.447 | | 3.939 | 1.135 | | 2.981 | | 16.9 | |
| virginiensis | | 1.547 | 2.025 | | 5.285 | 2.429 | | 2.952 | | 22.3 | |
| virginiensis | | 1.203 | 1.831 | | 4.739 | 2.313 | | 2.426 | | 15.3 | |
| virginiensis | | 1.35 | 2 | | 5.1 | 1.69 | | 2.685 | | 16.4 | |
| virginiensis | | 1.068 | 1.801 | | 4.167 | 1.466 | | 2.03 | | 22.1 | |
| virginiensis | | 1.25 | 2.04 | | 4.66 | 1.82 | | 2.828 | | 17.9 | |
| virginiensis | | 1.073 | 1.626 | | 3.499 | 1.33 | | 1.976 | | 9.7 | |
| virginiensis | | 1.413 | 1.593 | | 3.426 | 1.451 | | 2.06 | | 8.6 | |
| virginiensis | | 1.085 | 1.769 | | 3.749 | 1.386 | | 1.818 | | 20.6 | |
| virginiensis | | 1.36 | 1.59 | | 4.078 | 1.445 | | 2.545 | | 6.5 | |
| escarpment | | 1.2 | 2.89 | | 5.61 | 2.19 | | 3.56 | | 16 | |
| escarpment | | 1.14 | 2.99 | | 5.72 | 2.52 | | 3.13 | | 20.5 | |
| escarpment | | 1.15 | 3.27 | | 6 | 2.68 | | 4.53 | | 16 | |
| escarpment | | 0.78 | 2.62 | | 4.64 | 1.81 | | 3.79 | | 12.5 | |
| escarpment | | 1.11 | 2.88 | | 4.91 | 2.42 | | 3.82 | | 15 | |
| escarpment | | 0.89 | 2.32 | | 3.92 | 1.37 | | 3.75 | | 25 | |
| escarpment | | 0.87 | 2.59 | | 4.29 | 1.69 | | 2.98 | | 18.1 | |
| escarpment | | 0.9 | 2.65 | | 4.35 | 1.73 | | 4.42 | | 13.5 | |
| escarpment | | 1.36 | 3.12 | | 5.06 | 2.43 | | 4.935 | | 18.5 | |
| escarpment | | 0.98 | 3.17 | | 5.14 | 1.7 | | 3.74 | | 14.5 | |
| escarpment | | 1.03 | 2.83 | | 4.43 | 1.58 | | 3.86 | | 12 | |
| escarpment | | 1.13 | 3.57 | | 5.556 | 2.36 | | 4.6 | | 19.4 | |
| escarpment | | 1.32 | 3.54 | | 5.29 | 2.19 | | 5.34 | | 22.5 | |
| escarpment | | 0.7 | 2.24 | | 2.97 | 1.11 | | 2.19 | | 26 | |
| escarpment | | 0.893 | 2.81 | | 3.538 | 1.374 | | 4.78 | | 22.2 | |
| careyana | | 0 | 2.66 | | 4.4 | 1.69 | | 2.66 | | 15 | |
| careyana | | 0 | 2.65 | | 4.37 | 1.56 | | 1.99 | | 12.5 | |
| careyana | | 0 | 2.33 | | 3.73 | 1.63 | | 1.94 | | 12 | |
| careyana | | 0 | 2.6 | | 3.93 | 1.58 | | 2.08 | | 10.5 | |
| careyana | | 0 | 2.55 | | 3.79 | 1.55 | | 2.53 | | 8.5 | |
| careyana | | 0 | 2.64 | | 3.83 | 1.83 | | 1.93 | | 9 | |
| careyana | | 0 | 2.6 | | 3.64 | 1.54 | | 1.69 | | 12.5 | |
| careyana | | 0 | 3.23 | | 4.46 | 2.22 | | 2.84 | | 19 | |
| careyana | | 0 | 2.54 | | 3.4 | 1.58 | | 1.81 | | 12 | |
| careyana | | 0 | 2.557 | | 3.385 | 1.517 | | 2.345 | | 19.5 | |
| careyana | | 0 | 2.8 | | 3.58 | 1.67 | | 1.56 | | 6.5 | |
| careyana | | 0 | 3.466 | | 4.266 | 2.022 | | 2.968 | | 21 | |
| careyana | | 0 | 3.894 | | 4.638 | 2.194 | | 3.968 | | 20.7 | |
|  | |  |  | |  |  | |  | |  | |
| Species | Blade Length (cm) | | Blade Width (cm) | Petiole Length (cm) | | | Sqrt Blade Area (cm2) | | Blade Circularity | |  |
| virginiensis | 4.419 | | 2.444 | 4.37 | | | 2.912215651 | | 0.351 | |  |
| virginiensis | 1.171 | | 1.038 | 0.845 | | | 0.976729236 | | 0.43 | |  |
| virginiensis | 2.591 | | 1.686 | 1.035 | | | 1.852565788 | | 0.289 | |  |
| virginiensis | 1.277 | | 0.837 | 0.656 | | | 0.916515139 | | 0.433 | |  |
| virginiensis | 0.809 | | 0.759 | 1.344 | | | 0.694262198 | | 0.353 | |  |
| virginiensis | 3.249 | | 2.503 | 1.29 | | | 2.527449307 | | 0.407 | |  |
| virginiensis | 2.797 | | 1.183 | 0.478 | | | 1.564928113 | | 0.186 | |  |
| virginiensis | 1.746 | | 1.359 | 1.736 | | | 1.365283853 | | 0.412 | |  |
| virginiensis | 2.864 | | 1.402 | 2.215 | | | 1.775387282 | | 0.414 | |  |
| virginiensis | 4.08 | | 2.555 | 1.772 | | | 2.861642885 | | 0.359 | |  |
| virginiensis | 2.168 | | 1.538 | 1.476 | | | 1.618641406 | | 0.395 | |  |
| virginiensis | 2.658 | | 1.907 | 1.949 | | | 1.994993734 | | 0.4 | |  |
| virginiensis | 2.261 | | 2.099 | 2.993 | | | 1.930802942 | | 0.299 | |  |
| virginiensis | 2.89 | | 1.684 | 1.838 | | | 1.954993606 | | 0.299 | |  |
| virginiensis | 2.4 | | 0.524 | 8.64 | | | 0.643428318 | | 0.3733 | |  |
| virginiensis | 1.474 | | 0.886 | 0.487 | | | 1.012916581 | | 0.395 | |  |
| virginiensis | 1.128 | | 0.724 | 0.918 | | | 0.800624756 | | 0.349 | |  |
| virginiensis | 1.712 | | 1.045 | 0.475 | | | 1.185326959 | | 0.456 | |  |
| virginiensis | 2.849 | | 1.324 | 1.968 | | | 1.72133669 | | 0.358 | |  |
| virginiensis | 3.046 | | 2.119 | 1.644 | | | 2.25166605 | | 0.315 | |  |
| virginiensis | 2.435 | | 1.688 | 1.496 | | | 1.665232716 | | 0.418 | |  |
| virginiensis | 2.712 | | 1.552 | 1.379 | | | 1.818515878 | | 0.338 | |  |
| virginiensis | 2.67 | | 1.623 | 2.329 | | | 1.844722201 | | 0.473 | |  |
| virginiensis | 2.083 | | 1.039 | 1.634 | | | 1.280234354 | | 0.289 | |  |

Supplementary Table 5. *M. virginiensis* fruit measurements used in PCA. All measurements in cm.

| PCA Number | Specimen ID | Plant Height | Distance Between Fruit Horns | Fruit Length |
| --- | --- | --- | --- | --- |
| 1 | UNA00034416 | 21.7 | 0.475 | 0.285 |
| 2 | UNA00034491 | 25.49 | 0.516 | 0.285 |
| 3 | UNA00034496 | 22.848 | 0.555 | 0.271 |
| 4 | NCU00090765 | 26.391 | 0.43 | 0.382 |
| 5 | UNA00014924 | 15.017 | 0.355 | 0.294 |
| 6 | UNA00065282 | 27.698 | 0.463 | 0.241 |
| 7 | NCU00090772 | 15.18 | 0.45 | 0.282 |
| 8 | NCU00090770 | 17.741 | 0.405 | 0.39 |
| 9 | ANHC009866 | 29.181 | 0.553 | 0.362 |
| 10 | UVMVT068793 | 25.226 | 0.388 | 0.274 |
| 11 | NCU00090930 | 23.382 | 0.468 | 0.331 |
| 12 | PH00498081 | 33.845 | 0.673 | 0.549 |
| 13 | NCU00090787 | 16.045 | 0.449 | 0.285 |
| 14 | CLEMS0066960 | 26.651 | 0.367 | 0.332 |
| 15 | GA035914 | 19.886 | 0.557 | 0.339 |
| 16 | NCU00090982 | 8.687 | 0.42 | 0.398 |
| 17 | IND-0046733 | 30.121 | 0.384 | 0.311 |
| 18 | IND-0046741 | 29.015 | 0.589 | 0.321 |
| 19 | IND-0046743 | 17.95 | 0.338 | 0.276 |
| 20 | NCU00090791 | 16.21 | 0.479 | 0.326 |
| 21 | MARY1018304 | 18.361 | 0.503 | 0.371 |
| 22 | DOV0036330 | 14.006 | 0.388 | 0.306 |
| 23 | NCU00090907 | 18.31 | 0.483 | 0.386 |
| 24 | MARY1018308 | 22.681 | 0.343 | 0.352 |
| 25 | HUDC00009878 | 18.826 | 0.567 | 0.424 |
| 26 | MARY1018360 | 30.386 | 0.413 | 0.386 |
| 27 | UVMVT144783 | 16.179 | 0.413 | 0.333 |
| 28 | 4737 | 22.352 | 0.647 | 0.375 |
| 29 | IND-0046746 | 14.387 | 0.346 | 0.273 |
| 30 | 1465255 | 19.053 | 0.398 | 0.365 |
| 31 | 1465257 | 33.195 | 0.368 | 0.384 |
| 32 | 1477443 | 16.781 | 0.357 | 0.384 |
| 33 | 1465249 | 19.348 | 0.388 | 0.339 |
| 34 | 1465270 | 9.229 | 0.332 | 0.296 |
| 35 | 1465253 | 13.698 | 0.412 | 0.353 |
| 36 | UNCC_45631 | 20.592 | 0.4 | 0.313 |
| 37 | MMNS006411 | 23.363 | 0.343 | 0.395 |
| 38 | 59163 | 15.059 | 0.385 | 0.379 |
| 39 | ANHC010841 | 14.695 | 0.483 | 0.301 |
| 40 | NCU00090991 | 15.14 | 0.509 | 0.284 |
| 41 | UVMVT068805 | 13.367 | 0.467 | 0.311 |
| 42 | PH00498301 | 25.175 | 0.52 | 0.354 |
| 43 | PH00498241 | 24.849 | 0.42 | 0.266 |
| 44 | 1246908 | 21.645 | 0.506 | 0.339 |
| 45 | SIM0003763 | 24.807 | 0.459 | 0.559 |
| 46 | NCU00088179 | 39.099 | 0.411 | 0.395 |
| 47 | NCU00088161 | 16.1247 | 0.509 | 0.35 |
| 48 | NCU00088181 | 29.534 | 0.38 | 0.394 |
| 49 | NCU00088142 | 37.342 | 0.602 | 0.342 |
| 50 | NCU00088173 | 26.267 | 0.609 | 0.354 |
| 51 | NCU00088176 | 20.484 | 0.893 | 0.501 |
| 52 | NCU00088173 | 24.96 | 0.611 | 0.369 |
| 53 | NCU00088177 | 25.974 | 0.612 | 0.521 |
| 54 | NCU00090960 | 36.761 | 0.321 | 0.427 |
| 55 | PH00498115 | 15.836 | 0.591 | 0.38 |
| 56 | PH00497956 | 35.634 | 0.413 | 0.316 |
| 57 | PH00498196 | 32.488 | 0.435 | 0.361 |
| 58 | PH00498109 | 31.678 | 0.12 | 0.189 |
| 59 | PH00498199 | 24.095 | 0.493 | 0.317 |
| 60 | PH00498182 | 20.38 | 0.319 | 0.374 |
| 61 | PH00497914 | 27.901 | 0.317 | 0.286 |
| 62 | IND-0046755 | 16.412 | 0.319 | 0.292 |
| 63 | PBRU00056700 | 24.349 | 0.403 | 0.5 |
| 64 | CLEMS0067009 | 21.828 | 0.431 | 0.373 |
| 65 | USCH0057789 | 31.721 | 0.69 | 0.424 |
| 66 | CLEMS0067011 | 15.826 | 0.618 | 0.393 |
| 67 | CLEMS0067017 | 19.927 | 0.36 | 0.274 |
| 68 | CLEMS0067008 | 22.66 | 0.384 | 0.326 |
| 69 | CLEMS0067012 | 11.412 | 0.593 | 0.34 |
| 70 | NCU00090720 | 23.566 | 0.375 | 0.33 |
| 71 | APSC0003160 | 18.218 | 0.366 | 0.31 |
| 72 | NCU00090910 | 21.095 | 0.309 | 0.429 |
| 73 | WCUH0024320 | 23.48 | 0.426 | 0.3 |
| 74 | UVMVT068755 | 32.849 | 0.371 | 0.351 |
| 75 | UVMVT068741 | 15.735 | 0.515 | 0.334 |
| 76 | UVMVT068723 | 19.911 | 0.355 | 0.267 |
| 77 | UVMVT068708 | 26.677 | 0.263 | 0.298 |
| 78 | 18702 | 21.036 | 0.512 | 0.371 |
| 79 | NCU00092555 | 17.499 | 0.34 | 0.382 |
| 80 | 1400883 | 29.314 | 0.689 | 0.343 |
| 81 | WVA-V-0068747 | 16.935 | 0.451 | 0.46 |
| 82 | WVA-V-0025989 | 16.566 | 0.359 | 0.272 |
| 83 | WVA-V-0068718 | 25.504 | 0.45 | 0.447 |
| 84 | WVA-V-0068668 | 22.000 | 0.303 | 0.351 |
| 85 | WVA-V-0068728 | 31.526 | 0.548 | 0.332 |
| 86 | WVA-V-0068706 | 23.051 | 0.33 | 0.368 |
| 87 | WVA-V-0068654 | 16.038 | 0.6 | 0.35 |
| 88 | WVA-V-0068674 | 18.068 | 0.521 | 0.354 |
| 89 | WVA-V-0068669 | 18.592 | 0.532 | 0.29 |

Supplementary Table 6. Summary of PERMANOVA results from the correlation analysis of morphological measurements and geographic variables for *M*. *virginiensis* using somatic-reproductive allocation (PCA2) as response, and elevation, longitude, and their interaction, as predictors. Only the main effect of elevation was found statistically significant (*).

| Predictor | Df | Sum Sq | Mean Sq | F value | Pr(>F) |
| --- | --- | --- | --- | --- | --- |
| Elevation | 1 | 12.982 | 12.982 | 6.43 | *0.02 |
| Longitude | 1 | 1.186 | 1.186 | 0.587 | 0.452 |
| Elevation:Longitude | 1 | 1.675 | 1.675 | 0.83 | 0.373 |
| Residuals | 20 | 40.382 | 2.019 |  |  |
